# Gems from traditional north-African medicine: medicinal and aromatic plants from Sudan

**DOI:** 10.1007/s13659-012-0015-2

**Published:** 2012-04-17

**Authors:** Hassan Khalid, Wail Elsadig Abdalla, Haider Abdelgadir, Till Opatz, Thomas Efferth

**Affiliations:** 1 The Medicinal and Aromatic Plants Research Institute (MAPRI), National Centre for Research, Mac Nimr Street, Khartoum, Sudan; 2 Institute of Organic Chemistry, Johannes Gutenberg University, Duesbergweg 10-14, 55128 Mainz, Germany; 3 Department of Pharmaceutical Biology, Institute of Pharmacy and Biochemistry, Johannes Gutenberg University, Staudinger Weg 5, 55128 Mainz, Germany

**Keywords:** herbal medicine, natural products, pharmacognosy, phytochemistry, phytotherapy, traditional medicine

## Abstract

Sudanese folk medicine represents a unique blend of indigenous cultures with Islamic, Arabic and African traditions. In addition, Sudan encompasses different terrains and climatic zones, ranging from desert and semi-desert in the north to equatorial with a short rainy season (semi-aridand semi-humid) in the centre to equatorial with a long rainy season (arid-humid and equatorialhumid) in the south. This variation contributes to the immense diversity of vegetation in the region. The flora of Sudan consists of 3137 species of flowering plants belonging to 170 families and 1280 genera. It is estimated that 15% of these plants are endemic to Sudan. The intersection of diverse cultures and the unique geography holds great potential for Sudanese herbal medicine. Medicinal and aromatic plants and their derivatives represent an integral part of life in Sudan. Indigenous remedies are the only form of therapy available to the majority of poor people. It has been estimated that only 11% of the population has access to formal health care. Therefore, research on the desired pharmacological effects and possible unwanted side effects or toxicity is required to improve efficacy and safety of Sudanese herbal medicine. In the future, it would be preferable to promote the use of traditional herbal remedies by conversion of raw plant material into more sophisticated products instead of completely replacing the traditional remedies with synthetic products from industrialized countries. The present review gives an overview of traditional Sudanese medicinal and aromatic herbs and their habitats, traditional uses, and phytochemical constituents.

**Electronic Supplementary Material:**

Supplementary material is available for this article at 10.1007/s13659-012-0015-2 and is accessible for authorized users.

## 1 Historical Roots of Medicine in Sudan

*Global dimension of traditional medicines*

Traditional medicines are used widely throughout the world. As the name implies, these treatments are a part of the traditions of each country that have been handed down from generation to generation. Acceptance of traditional medicines by a population is largely conditioned by cultural factors.

Acknowledging the potential value of traditional medicine for the expansion of health service, the World Health Assembly (WHA) passed a number of resolutions in 1976 to draw attention to the potential reserve constituted by traditional practitioners. In 1977, WHA urged countries to utilize their traditional system of medicine. In 1979, WHA called for a comprehensive approach to the subject of medicinal plants. The 40$^{th}$ WHA resolution (1987) reaffirmed the main points of earlier resolutions and recommendations made by the Conference on Primary Health Care Systems convened by the World Health Organization (WHO) and The United Nations Children's Fund (UNICEF) in Alma Atta, USSR in 1978. These resolutions gave the organization a fresh mandate of future action in this field. It specifically urged member states to initiate comprehensive programmes for the identification, evaluation, preparation and conservation of medicinal plants used in traditional medicine and to ensure quality control with respect to drugs derived from traditional plant remedies by using modern techniques and applying suitable standards and good manufacturing practices.

In March 1988, an international consultation on the conservation of medicinal plants was convened in Chaing Mai, Thailand by the WHO in association with the International Union for Conservation of Nature and Natural Resources (IUCN) and the World Wildlife Fund (WWF). One outcome of this meeting was the adoption of the Chaing Mai Declaration entitled "Save Plants Save Nature". Two months later, in May 1988, the 41$^{st}$ WHA endorsed the Chaing Mai Declaration and its call for international co-operation and coordination in

*To whom correspondence should be addressed. E-mail: efferth@uni-mainz.de establishing conservation programmes to ensure availability of adequate quantities of medicinal plants for future generations.[1,2]

*Historical pioneers of traditional medicine*

In the ancient Egyptian civilization (1500 B.C.), people believed that diseases were initiated by magicians using magic practices. Asclepiad and his Egyptian priests usually advised people to sleep to treat diseases. In the 16th century, a new system came over from India known as Ayurveda. With a strong relation to Buddhism, Ayurvedic practices mainly depend on elements such as air, water, soil and fire controlling the world. The system views the body as formed of lymph, blood, fats, and bones. Accordingly, health is a result of the equilibrium between these elements and any medicinal treatment restores or maintains this equilibrium.

During the Roman Empire, more attention was paid to public health. Sport and hygiene as preventive measures were more in the focus of public interest than actual treatment with medications such as herbs etc.

In the 2nd century, the medicinal school of Galen (131–201) focused on treatment. Galen believed that everything in nature has a goal or target. He performed many experiments on animals and extensively researched recipes with natural products and medicinal plants. Galenicals, medicines prepared by Galen's procedures, are still considered an important discipline in modern pharmacy.

The Islamic and Arabic civilizations have had a great impact on medicinal knowledge. These civilizations performed experimental studies and developed ethical codes, new methods of preparing and dispensing pharmaceuticals and novel treatment strategies. This is comparable in a sense to modern pharmaceutical sciences.

The first pharmacy was established in Bagdad in the years 750–754. In Europe in 1240, Fredrick II decreed that pharmacy be separated from medicine. A second historical milestone was the regulation of pharmacy practice and code of ethics developed by Alkhalifa Al Mugtadir (908–938).[3] He applied the code of ethics based on the pharmacopeia of Gallen and Hanin Isak, which described adulterations and their detection.

Islamic and Arabic civilizations also contributed to drug treatment strategiesstill used today. For example, Majousy (died in 944) reported in his book *Complete Medical Management* that "If you can treat a patient with diet, do not prescribe a drug, and if you can treat with one simple drug, do not use a complex drug and do not use strange or unknown drugs". Abu Bakr Al Razi (854–932) stated in his book *Al Hawi*[4] "If a Physician treats a disease with diet and nutrition, then he is successful and happy". This book represents a huge encyclopaedia of 12 sections. Today, some of these sections are scattered around European museums, while others have been lost.

At that time, physicians used a number of medicinal plants such as Indian Snakeroot (*Rauwolfia serpentina*), Black Cumin (*Nigella sativa*.), *Senna alexandrina*, Myrrh (*Commiphora myrrah*) and others. They knew about anesthesia and the epidemiology of leprosy and anthrax. Ibn El Bitar(1248 A.D.)[5] reported more than 1400 drugs and their sources, benefits and side effects. Important pioneers in the history of botany and pharmacy include Ibn El Bitar,[5] Al Dainori (1953),[6] Daoud Al-Antaky (1600 A.D.),[7] with his famous book Altazkira, Ibn Alawam and others.

*Traditional African medicine*

Traditional African medicine is an ancient holistic system of healing that makes extensive use of indigenous herbs combined with African spirituality. It continues to thrive in Africa today, and practitioners can also be found in many other parts of the world.[8] Under colonial rule, many nations considered traditional spiritual healers to be practitioners of witchcraft and outlawed them for that reason. Many different forms of traditional medicinal practices have evolved against widely different ethnological, cultural, climatic, geographical, and even philosophical backgrounds. In many of these practices, diviners are responsible for determining the cause of illnesses, which in some cases is believed to stem from ancestral spirits and other supernatural influences. Traditional African medicine involves diviners, midwives, herbalists and other types of practitioners.

Practitioners of traditional African medicine claim to be able to cure a wide range of conditions, including cancer, acquired immunodeficiency syndrome (AIDS), psychiatric disorders, high blood pressure, cholera, infertility, and most venereal diseases.

*Traditional Sudanese medicine*

Sudanese folk medicine represents a unique blend of indigenous cultures of Islamic, Arabic and African traditions. Consequently, treatments exist for a variety of diseases, both epidemic and endemic. To face these diseases, people have tapped the environmental resources, *e.g.* plants, minerals and animal products for the management of their health.[9–13] In this respect, the Sudanese have amassed a large body of curative methods, techniques and recipes. Readers may find further recent and detailed information in the descriptive inventory, which appeared in the Atlas of Medicinal Plants series published by Medicinal and Aromatic Plants Research (MAPRI). This series includes comprehensive surveys of the medicinal plants Erkawit, Nuba Mountains, White Nile, North Kordofan, and Angasana.[14–18]

Though not yet investigated systematically or in depth, there are clues in literature about the bioactivity of medicinal plants and their chemical constituents. Sudanese medicinal plants have been reported to exert antimicrobial activity against viruses, bacteria, and protozoa.[19–23] As infections with worms or molluscs represent a common affliction in that area, medicinal plants have been considered for treatment of these infections.[24–27] Immunomodulatory properties of Sudanese medicinal plants have also been observed.[28] In this review, we provide an updated overview of the most important plants used in Sudanese traditional medicines.

## 2 Geography, Vegetation Regions, and Medicinal Plants of Sudan

Sudan is the largest country in Africa with an area of approximately one million square miles. This area lies between latitudes 4° N and 22° N and longitudes 22° E and 38° E. Such an area, with its diverse climatic conditions and different ethnic communities, holds the potential of an immense wealth of flora with a variety of uses in traditional medicine. Many of these plants have been used by herbalists for different purposes.

Sudan encompasses different terrains and climatic zones, ranging from desert and semi-desert in the north to equatorial with a short rainy season (semi-arid and semi-humid) in the centre to equatorial with a long rainy season (arid-humid and equatorial-humid) in the south. Thus, the range of mean annual rainfall (m.a.r.) is expected to vary in accordance with the climatic zones. It ranges from zero to heavy (1400 mm). Depending on the climatic zones and the amount of rainfall, the type of soil and cultivation practices are usually classified as one of four categories ranging from humid and rainy climate (Ferrasols and Nitosols types) to arid climate (Yermosols poor soil types). This climatic variation has a direct impact on the immense diversity of vegetation. Based on the variation of climate, White (1983)[29] divided Sudan into five main vegetationregions:

1. Desert.
2. Semi-desert scrub and grassland.
3. Thorn savanna and scrub.
4. Deciduous savanna woodland.
5. Flood region.

## 3 Overview of Medicinal Plants in Sudan

The flora of Sudan consists of 3137 documented species of flowering plants belonging to 170 families and 1280 genera. It is estimated that 15% of these plants are endemic to Sudan. The intersection of cultures and the unique geographical position of Sudan hold great potential for research in many fields, the most important of which is medicinal and aromatic plants. The diversity of climates in Sudan results in a rich variety of flora species corresponding to the wide range of ecological habitats and vegetation zones. An overview of Sudanese medicinal plants from these regions is shown in Table I.

In Sudan, it is a common practice to collect medicinal plants from their natural habitats for home consumption and export. Plants collected from different localities or geographic regions may have different chemical compositions. This may be explained by differences in climate, temperature, rainfall, altitude, day length and UV-radiation, all of which play an important role in plant development and affect the biosynthesis of secondary metabolites with biological activity. In general, generation of volatile oils appears to be enhanced at higher temperatures. Continuous rainfall can lead to a loss of water-soluble substances from leaves and roots by leaching and also makes collection and drying more difficult. For example, when peppermint is grown in Sudan under long-day conditions, the leaves contain high amounts of menthone and menthol with only trace amounts of menthofuran, whereas peppermint plants grown under short-day conditions contain menthofuran as the major component. The amount of bitter constituents in *Gentiana lutea* increases with altitude, whereas alkaloid contents in*Aconitum napellus* and *Lobelia inflata* and essential oils in thymus peppermint decrease. *Pyrethrum* delivers the best yield of pyrethrins when cultivated near the equator and at high altitudes.[30]

## 4 Use of Medicinal and Aromatic Plants in Traditional Medicine in Sudan

Due to the tremendous wealth of plants in Sudan as well as external influences on traditional medical practices from neighbouring countries and different Sudanese ethnicities, Sudan is a melting pot of medical traits, especially those concerning herbal medicine.

Like many other countries, Sudan has a long tradition of folk medicine. Indigenous remedies are often the only form of therapy available to poor people living in rural areas or cities (Table II). It has been estimated that only 11% of the population has access to formal health care. The governmental medical services, which were free of charge in the past, are now available at high charges. Poor people frequently cannot afford medicines. Since medical care is not available or is too expensive, the majority of people depend on traditional remedies prescribed by unregistered traditional healers.[31]

Medicinal and aromatic plants and their derivatives represent an integral part of the life and culture of the people of Sudan. This is not only true for treatment of human diseases, but also for veterinary use (Table III).[32,33] Because of the tremendous importance of medicinal plants for human and animal health, research on valuable pharmacological effects and possible unwanted side effects or toxicity is required to improve the efficacy and safety of Sudanese herbal medicine. Plants produce secondary metabolites as defence weapons against predators such as microbes or herbivores. Hence, it comes as no surprise that plants do produce poisonous substances. Therefore, valuable pharmacological activities of medicinal plants have to be distinguished from toxic plant effects.[32,33–38] The Sudanese society has become more affluent and sophisticated with the country's economic growth. In the future, it would be preferable to promote the use of traditional herbal remedies by conversion of raw plant material into more sophisticated products instead of completely replacing the traditional remedies with synthetic products from industrialized countries. As an example, Muswak *(Salvadora persica)* stem is used to clean teeth by brushing. This plant could be developed as tooth paste in the future. Senna leaves and pods (*Senna alexandrina*) are commonly boiled and used as a purgative. Argel leaves (*Solnostemma argel*) are traditionally boiled and drunk by women for menstrual disorders. Haza herb (*Haplophyllum tuberculatum*) is boiled and used against diarrhoea and spasms. Henna leaves (*Lawsonia inermis*) are crushed to produce a dye for decorative patterns on the palm, hands and feet of women or used as a hair dye.

The traditional medical practitioners in Sudan have no formal training or pharmacopoeia, and their knowledge is based on acquired folklore and indigenous traditions. It cannot be compared with the Sri Lankan and Indian Ayurvedic, Yonani, or traditional Chinese medical systems, where formal training is provided.

Sudanese people use an incense mixture called *bakour* which is composed of dried plant material, gum, exudates, and balsam. When burnt in a traditional clay incense burner, it gives a fragrant smoke, which is believed to clean air, repel insects and make the environment smell pleasant.

Sudan has a unique tradition of preparing numerous different local perfumes. The basic compounds in these perfumes are *Khumra,* which is prepared from *mahleb* seeds (*Prunus mahleb*), Sandalwood powder (*Santalum album*) and different brands of known perfumes, lotions and *eau de colognes*. *Khumra* consists of sixteen ingredients. *Khumra* can be classified as *Khumra zeit* (oil-based), *Khumra dufra* (includes shells), or *Khumra misk* (contains extracts of crocodile's sweat glands). During the sophisticated production of the *ajina*

**Table I. Medicinal plants from different vegetative regions of Sudan and their medicinal uses**

| Latin name | Local name | Family | Habit | Medicinal use |
|---|---|---|---|---|
| **Common medicinal plant species of the desert** | | | | |
| *Boscia senegalensis* (Pers.) L+am. | Kursan; Mukheit | Capparaceae | Shrub/ Small tree | Anti-rheumatic, against gonorrhea and urinary tract inflammations[16]; Anthelmintic, eye wash;[17] against tuberculosis[15] |
| *Cadaba farinosa* Forssk. | Surreh | Capparaceae | Tomentose shrub/small tree | Taenifuge, gargeled for tonsillitis[15] |
| *Fagonia cretica* L. | Umm Shuwaika | Zygophyllaceae | Spinescent glabrous herb | Against muscular pains;[16] Antispasmodic, antipurgative;[17] against heart burn[14] |
| *Ziziphus spina-christi* L. | Sidir | Rhamnaceae | Spinescent tree | Antispasmodic, against gonorrhea;[15,16] anti-purgative,[17] |
| **Common medicinal plant species of the semi-desert** | | | | |
| *Acacia oerfota* (Forssk.) Schweinf. | La'ot | Fabaceae (Mimosoideae) | Spinescent shrub | Anti-rheumatic;[17] against snake, against swelling & scorpion bites[15,16] |
| *Acacia senegal* (L.) Willd. | Hashab | Fabaceae (Mimosoideae) | Spinescent small tree | For stomach ulcers, anti-diarrheal[18] |
| *Balanites aegyptiaca* (L.) Del. | Higleeg | Balanitaceae | Spinescent tree | Purgative;[16,17,18] wound healing;[16] anti-rheumatic;[14] anti-diabetic[17]; anthelmintic[15,16] |
| *Calotropis procera* (Ait.) Ait.f. | Ushar | Asclepiadaceae | Ashy woody shrub | Against scorpion bites and jaundice [15]; healing thorn injuries[17]; anti-rheumatic [18] |
| *Capparis decidua* Forssk. | Taundub | Capparaceae | Spinescent glabrous deciduous shrub | Against jaundice [14,15,17]; to treat swelling [15]; against headache [16]; anti-rheumatic [15,17] |
| *Leptadenia pyrotechnica* (Forssk.) DC. | Marakh | Asclepiadaceae | Glabrous mostly leafless shrub | Anti-rheumatic [17,18] diuretic[17] |
| *Maerua crassifolia* Forssk. | Sarah | Capparaceae | small pubescent evergreen tree | Anti-rheumatic[17] |
| *Salvadora persica* L. | Arak | Salvadoraceae | small evergreen tree | Detergent for mouth, facilitates digestion |
| *Senna alexandrina* Mill. (*Cassia senna* L.) | Senna Mekka | Fabaceae (Caesalpinioideae) | Glabrous | Laxative[14,18]; against gastro-intestinal disorders "GID"[18] |
| *Ziziphus spina-christi* L | Sidir | Rhamnaceae | Spinescent tree | Antispasmodic[15,16]; anti-purgative[17] against gonorrhea[15] |
| **Common medicinal plant species of the thorn savanna and scrub** | | | | |
| *Acacia senegal* (L.) Willd. | Hashab | Fabaceae (Mimosoideae) | Spinescent small tree | Against stomach ulcers, anti-diarrheal[18] |
| *Acacia seyal* Del. | Talih | Fabaceae (Mimosoideae) | Spinescent tree | Anti-rheumatic, mouth detergent[18] |
| *Albizzia amara* (Roxb.) Boiv. | Arrada | Fabaceae (Mimosoideae) | Deciduous tree | Against jaundice, mouth inflammation and chest pain[15] wound healing[17] |
| *Balanites aegyptiaca* (L.) Del. | Higleeg | Balanitaceae | Spinescent tree | Purgative[15,16,17]; wound healing[16]; anti-rheumatic[14], anti-diabetic[17]; anthelmintic[15,16] |
| *Cadaba glandulosa* Forssk. | Kurmut | Capparaceae | Glandular many-branched shrub | Against swelling[14,15,17]; anti-rheumatic[16] |
| *Calotropis procera* (Ait.) Ait.f. | Ushar | Asclepiadaceae | Ashy woody shrub | Anti-rheumatic, mouth detergent[18] |
| *Dalbergia melanoxylon* Guill. & Perr. | Abanous | Fabaceae (Papilionoideae) | Deciduous multi-stemmed tree | Anti-rheumatic[15]; against heart pain[16] |
| *Sclerocarya birrea* (A.Rich.) Hochst. | Hemaid | Anacardiaceae | Ashy glabrous tree | Anti-spasmodic, anti-diarrheal[15]; Anthelmintic; wound healing[18] |

| | | | | |
|---|---|---|---|---|
| **Common medicinal plant species of the deciduous savannah** | | | | |
| *Acacia seyal* Del. | Talih | Fabaceae (Mimosoideae) | Spinescent tree | Anti-rheumatic, mouth detergent[18] |
| *Albizzia amara* (Roxb.) Boiv. | Arrada | Fabaceae (Mimosoideae) | Deciduous tree | Against jaundice, mouth inflammation and chest pain[15]; wound healing[17] |
| *Combretum hartmannia-num*Schweinf. | Habeil | Combretacea | Glabrous medium-sized tree | Against jaundice[15,17] |
| *Khaya senegalensis*(Desr.) A. Juss. | Mahogany | Meliaceae | Deciduous tree | Anti-malarial, against hepatic inflamma-tion, sinusitis, skin diseases, GID and trachoma[18] |
| *Terminalia brownie* Fresen. | Sobagh, Shaff | Combretaceae | Tree | Against cough and bronchitis[17]; anti-rheumatic[18] |
| **Common medicinal plant species of the flood region** | | | | |
| *Acacia polyacantha* Willd. | Abu Sineina | Fabaceae (Mimosoideae) | Spinescent tree | Against jaundice and bilhariasis[15] |
| *Acacia seyal* Del. | Talih | Fabaceae (Mimosoideae) | Spinescent tree | Against jaundice[15,16] |
| *Balanites aegyptiaca* (L.) Del. | Hegleeg | Balanitaceae | Spinescent tree | Anti-rheumatic mouth detergent |
| *Combretum* sp. | Habeil | Combretaceae | Glabrous medium-sized tree | Purgative[15,16,17]; wound healing[16]; anti-rheumatic[14]; anti-diabetic[17] anthelmintic[15,16] |
| *Hyphaene thebaica* (L.) Mart. | Doum | Arecaceae (Palmae) | Tree | Against splenomegaly, alimentary system disorders and bacterial eye infections[18] |

**Table II. The most important plants used in traditional Sudanese medicine. Selected examples of isolated compounds from these plants are compiled in Electronic Supplementary Material.**

| | Latin Name | Local name | Folkloric Uses | Reference |
|---|---|---|---|---|
| 1. | *Acacia nilotica* (L.) Willd. ex Del. (Fabaceae, subfamily: Mimosoideae) | Garad, Sunt | The fruits are burned and the fumes are inhaled to treat colds and pharyngitis. The fruit macerates are used as anti-septic. | 43,44,45 |
| 2. | *Acacia senegal* (L.) Willd. (Fabaceae, subfamily: Mimosoideae) | Hashab Samug Arabi | Stem exudates (gums) are used as demulcents and against diarrhoea and ulcers. | 46 |
| 3. | *Acacia seyal* Del. (Fabaceae, subfamily: Mimosoideae) | Talih | Stem fumigant is used against rheumatic pain. | 47,48 |
| 4. | *Adansonia digitata* L. (Bombacaceae) | Tabaldi Gongelez | The fruits are used as a cold beverage, andare added to yoghurt for treatment of diarrhoea and amoebic dysentery. | 49 |
| 5. | *Aloe sinkatana* Rey. (Liliaceae) | Sabar | The leaf juice is used in treatment of skin diseases, constipation, anthelmintic and haemorrhoids. The leaves are also used to treat fever, diabetic, tonsillitis and inflammation of the colon. | 50 |
| 6. | *Ambrosia maritima* L. (Asteraceae) | Damsisa | The herbs are used in treatment of urinary tract infections and elimination of kidneystones, whereas the leaves are used as anti-diabetic and anti-hypertensive. | 51 |
| 7. | *Azadirachta indica* J.Juss. (Meliaceae) | Neem | The leaves are used to treat skin diseases, helminthiasis and malaria. | 52,53 |
| 8. | *Balanites aegyptiaca* (L.) Del. (Balanitaceae) | Heglig , Laloub | Fruits are uses to treat dysentery and constipation. The seed oil is used to treat tumors and wounds. | 54 |
| 9. | *Boscia senegalensis* (Pers.) Lam. ex Poir. (Capparaceae) | AL Khirusan | The roots are used to treat bilharzia, the leaves are used as a poultice for muscular pains and the fruits are used in treatment of tuberculosis. | 55 |
| 10. | *Boswellia papyrifera* (Del.) Hochst. (Burseraceae) | Luban | Manufacture of incense, an ingredient in plasters and pastilles. | 56 |
| 11. | *Capsicum frutescence* L. (Solanaceae) | Shata | The fruits are used as a food spice and stomachic whereas the fruit macerate is used as anti-rheumatic. | 57 |
| 12. | *Citrullus colocynthis* (L.) Schard. (Cucurbitaceae) | Handal | The fruits are used as anti-diabetic, purgative and for making tar, which is used as an anti-scabies treatment. | 17,58 |
| 13. | *Croton zambesicus* Mull-Arg. (Euphorbiaceae) | Habat El Malook | The fruits are used as an anti-malarial treatment. | 59,60,61 |

| | | | | |
|---|---|---|---|---|
| 14. | *Cucurbita maxima* L. (Cucurbitaceae) | Garah Aslai | The seeds are used as an anthelmintic and to treat skin diseases. | 62 |
| 15. | *Cymbopogon commutatus* (Poaceae) | | | 63 |
| 16. | *Cymbopogon nervatus* (Poaceae) | Nal | Antispasmodic | |
| 17. | *Cymbopogon proximus* (Hochst. ex A. Rich) Stapf. (Poaceae) | Mahareb | The leaves are used to treat gout, renal colic, helminthiasis, and inflammation of the prostate. | 64,65 |
| 18. | *Grewia tenax* ( Forssk.) Fiori. (Tiliaceae) | Godeim | The fruits are used to treat malaria and iron deficiency anaemia. | 66 |
| 19. | *Guiera senegalensis* J.F.Gmel. (Combretaceae) | Gubeish | The leaves are used as anti-hypertensive and anti-diabetic. | 67,68,69 |
| 20. | *Hibiscus sabdariffa* L. (Malvaceae) | Karkade Angara | The sepals are used as hot and cold beverages and to treat hypertension, colds, and fever and with other plants to treat malaria. | 70,71 |
| 21. | *Khaya senegalensis* (Desr.) A. Juss. (Meliaceae) | Mahogny | The stem bark is used to treat malaria, hepatic inflammations and enteriogasteritis. The leaves are used to treat skin diseases and trachoma. | 72,73 |
| 22. | *Lawsonia inermis* L. (Lytheraceae) | Henna | The leaves are used as anti-pyretic, for treatment of urinary tract infections, skin diseases and alopecia. | 74 |
| 23. | *Nauclia latifolia* Sm. (Rubiaceae) | Karmadoda | The roots are used for dysentery and as a tonic whereas the bark is used for abdominal colic. | 75,76 |
| 24. | *Ocimum basilicum* L. (Lamiaceae). | Rehan | The leaves and stems are used in treatment of jaundice, ascitis, spasm, parturition pain and scorpion stings. | 77 |
| 25. | *Phoenix dactylifera* L. (Palmae/Arecaceae) | Balah, Tamur | The fruits are used to treat constipation branchial asthma and tonsillitis. The pollen grains are mixed with bee-honey and ginger to increase fertility. | 78 |
| 26. | *Ricinus communis* L. (Euphorbiaceae) | Khirowi | The seed oil is used as a purgative and hair tonic and the whole seed is ingested as a contraceptive. The leaves are used as a poultice in treatment of abscesses. | 79,80 |
| 27. | *Salvadora persica* L. (Salvadoraceae) | Arak | The fruits are used to treat hypertension, stomach-pain and wounds, whereas the leaf-fibers are used to treat eye-infection. | 81,82 |
| 28. | *Senna alexandrina* Mill. (Fabaceae, subfamily Caesalpinioideae) | Sena Maca | The fruits are used as laxative and to treat gastroenteritis. | 83,84,85 |
| 29. | *Sesamum indicum* L. (Pedialiaceae) | Simsim | The seed oil is used alone or in combination of other drugs to treat tumours, skin diseases, alopecia, cough, colds, tonsillitis and fever. | 86,87 |
| 30. | *Solanum nigrum* L. (Solanaceae) | Inab Eldeeb | The fruits are used for gastroenteritis and colic pain. | 88 |
| 31. | *Solenostemma argel* (Del.) Hayne (Asclepiadaceae) | Hargel | The leaves are used as an antispasmodic, carminative and as an anti-diabetic. | 89,90 |
| 32. | *Tamarindus indica* L. (Fabaceae, subfamily Caesalpinioideae) | Ardeb | The fruits are used in treatments of constipation, malaria and jaundice. | 91 |
| 33. | *Terminalia brownii* Fresen. (Combretaceae) | Shaf | The stem and branch fumigants are used to treat rheumatic and back pains. | |
| 34. | *Trigonella foenum-graceum* L. (Fabaceae, subfamily Papilionoideae) | Hilba | The seeds are used as antidiarrheal, anti-spasmodic, anti-amoeba dysentery and anti-diabetics. The seeds are also used as a food additive and to increase secretion of lactating mothers and to facilitate expulsion of the placenta. | 92,93 |

**Table III. Plants used in veterinary medicine.**

| Plant | Part Used | Disease |
|---|---|---|
| *Acacia albida* (Fabaceae, subfamily: Mimosoideae) | Bark | Goat diarrhea |
| *Acacia nilotica* (Fabaceae, subfamily: Mimosoideae) | Fruit | For rinderpest / diarrhea |
| *Albizzia anthelmintica* (Fabaceae, subfamily: Mimosoideae) | Bark | Helmintic diseases |
| *Albizzia sericocephala* (Fabaceae, subfamily: Mimosoideae) | Bark | Swollen limbs |
| *Anogeissus schimperi* (Combretaceae) | Bark | |
| *Artemisia herba-alba* (Asteraceae) | Herb | Spasms and intestinal infections |
| *Balanites aegyptiaca* (Balantiaceae) | Fruit | Eye infections, trypanosomal infections |
| *Bauhinia* sp. (Fabaceae, subfamily Caesalpinioideae) | Leaves | Curl of limbs |

| | | |
|---|---|---|
| *Cadaba farinosa* (Capparaceae) | Leaves | Flatulence |
| *Cissus quadrangularis* (Ampelidaceae) | Whole plant | Uterus post abortion, wounds |
| *Cordia ovalis, C. rothii, C. sinensis* (Boraginacae) | Rootand stem bark | Itchy skin, retained placenta |
| *Citrullus coloynthis* (Cucurbitaceae) | Tar from seeds | Scabies |
| *Cucurbita pepo* (Cucurbitaceae) | Seeds | Helmintic diseases |
| *Cuminum cyminum* (Ranunculaceae) | Fruit | Spasms and intestinal infections |
| *Cymbopogon nervatus* (Poaceae) | Oil | Ectoparasites |
| *Diospyros mespiliformis* (Ebenaceae) | Bark | Aloric - diarrhea |
| *Euphorbia sp.* (Euphorbiaceae) | Whole plant | Deny (calf ill, bloat) |
| *Heliotropium strigosum* (Boraginaceae) | Whole plant | Helmintic diseases |
| *Gardenia lutea* (Rubiaceae) | Fruit | Constipation, bloat in calves |
| *Ipomoea aquatica* (Convolvulaceae) | Leaves | Against lice |
| *Maerua angolensis* (Capparaceae) | Roots | Bloat and pneumonia |
| *Nauclea latifolia* (Rubiaceae) | Flowers and Bark | Swollen knees |
| *Peganum harmala* (Zygophyllaceae) | Fruit | Endoparasites |

perfume (a paste made of various powdersof dura (*Sorghum* sp.) or Millet (*Pennisetum* sp.), the paste is smoked on a charcoal fire with pieces of sandalwood and gum olibanum.

There is also a famous traditional massage paste known as *Dilka*. The massage is usually carried out after anointing the body with oil which is scented with *Karkar*.[39]

*Dukhan* is another popular custom especially favoured by Sudanese women for sexual enhancement, cleanliness and also medical purposes. In this custom, Sudanese women use the smoke from Habil wood (*Combretum ghasalense*), Subakh (*Terminalia brownii*) and Talih (*Acacia seyal*) to fumigate their naked bodies while covering themselves with a sheet of cloth in the form of a tent. The main purpose is to impart fragrance into the skin by long exposure to the smoke of fragrant woods.[39,40]

## 5 Traditional Uses and Chemical Constituents of Sudanese Medicinal Plants

**Gum Arabica** is the dried exudation from the stem and branches of *Acacia senegal* (Leguminosae/Fabaceae). This thorny tree both grows in the wild and is cultivated in Sudan as well as west and central Africa. Habitat: The plant grows in sandy and clay-like soil in plains and short grass savannas, forming a continuous belt from east to west in central Sudan. It is very common in the western sand plains of Kordofan and Darfur. Constituents: The dried gum consists almost entirely of magnesium, calcium and potassium salts of glycosidal acid, known as Arabic acid, and also the enzyme oxidase, but has no or very few starch granules. Traditional use: Gum Arabica is used as binder and emulsifying agent, as well as an agent to increase the viscosity of pharmaceuticals and cosmetics. Furthermore, it is an ingredient in soft drink syrups, gummy candies and sweets.

**Aloes:** *Aloe spp*. varieties (Xanthorrhoeaceae/Aloaceae) grow in the wild in Sudan. *Aloe crassipes* is common in northern and eastern Sudan, while *A. sinkatana* is common in the east (Erkawit). Habitat: Aloes grow on sandy lowland plains of semi deserts and the Red Sea hills. Constituents: The plants containanthraquinone derivatives (Aloin). *Aloe emodi*also contains the glycoside barbolin. Traditional use: The dried mucilaginous substance obtained from leaves is used in small doses as a laxative.

**Balanite:** Heglig (*Balanites aegyptiaca* (L.) Del., Balantiaceae) is a semi-desert tree growing in the wild in south Kordofan, Darfur and Central Sudan. Habitat: This is a prevalent plant, which is common on the dark cracking clays of central Sudan.It is often associated with *Acacia seyal* on short grass savannas. Constituents: The root contains steroidal sapogenins, whereas the bulb contains sugars and saponins. The leaves and fruit contain diosgenin while the kernel with a high oil contents and valuable protein combination. Traditional use: The maceration of the fruit and seeds is used as a laxative and anthelmintic. It is used in food industry, for animal feed and in the pharmaceutical industry as a precursor in production.

**Basil:** *Ocimum basilicum* L. (Labiatae/Lamiaceae). Basil is an annual herb native to Asia and the Middle East and is common in Sudan. Habitat: Basil grows in the wild and isalso cultivated in northern and central Sudan, especially near streams. Constituents: Volatile oil containing cineol, pinene, methyl chavicol, d-camphor and ocimene. Traditional use: The essential oil obtained by distillation is used in perfumery and in food industry as aromatic and flavouring agent.

**Bitter Apple:** *Citrullus colocynthis* L. (Cucurbitaceae) is a monoecious, perennial vine, abundant in Asia and Africa. It grows in the wild in Sudan. Habitat: Bitter apple grows on sandy soil in the semi desert. The ripe fruits are collected in autumn, and deprived of their seeds and epicarp by peeling. Constituents: The plants contain an alkaloid, amorphous resin, a crystalline alcohol, citrullal, the glycoside of cucurbitin, oils and bitter principles. Traditional use: Bitter apple is used as a gastrointestinal stimulant or irritant. It is also a powerful purgative acting as a hydrogogue cathartic. In folk medicine, bitter apple is used as anti-rheumatic.

**Camels hay:** *Cymbopogon proximus* Stapf. (Poaceae) is an ascending, densely tufted perennial grass common in northern and central Sudan. Habitat: It is a perennial herb, grown in variable soils in moist areas during the rainy season. It is maintained as perennial plant in irrigated areas. Constituents: A bitter oleo resin, a toxic volatile oil and a saponin. Traditional use: It is extensively used as folk medicine to promote diuresis, to alleviate colic pain and as antipyretic plant against fever.

**Datura:** *Datura* species (*D. stramonium*, *D. metel*, *D. innoxia,* Solanaceae) are annual herbs native to North America that have been introduced to other parts of the world. *Datura*is wide-spread in Sudan. Habitat: Grows as a weed in northern and central Sudan on a wide range of soils throughout the year. Constituents: *Datura* plants contain alkaloids such as hyoscine and hyoscyamine, as well as atropine. Traditional use: it is a source of commercial hyoscyamine.

**Egyptian henbane:** (*Hyoscyamus muticus* L., Solanaceae) is a perennial herb grown in northern Sudan. Habitat: It grows in Sudan in a wide range of soils. It is sown all over the year. Constituents: Hyoscyamine, atropine and hyoscine. Traditional use: It is used as cerebral and spinal sedative.Egyptian henbane is also applied as purgative and is used against colic.

***Eucalyptus* spp.:** *Eucalyptus globulus*, (Myrtaceae) it is one of the tallest trees known. It is native to Australia and is cultivated in Italy, Spain, Algeria and Egypt. Habitat: It grows in various soils. It has been planted in many locations in Sudan. Constituents: *Eucalyptus* contains 3 to 6 % volatile oils. *Eucalyptus* oil contains not less than 50 % cineole (euclyptol) and also tannin and a bitter principle. Traditional use: The leaves are used as an astringent in the form of cigarettes against asthma. The oil exerts antispasmodic, deodorant and counter-irritant effects.

**Olibanum:** Luban (*Boswellia papyrifera,* Burseraceae) isa small tree thatcontains schizogenous ducts in the bark with a resin known as olibanum or frankinscence. Habitat: Luban grows on rocky ground in high-rainfall regions of the savanna of Blue Nile, Gebel Garri, Upper Nile and Equatoria. Constituents: Luban contains resin, gum, and volatile oil. Traditional use: It is used to manufacture incense and as an ingredient in plasters and pastilles.

**Sunt:** (*Acacia nilotica* (L.) Willed.ex Del., Fabaceae, subfamily Mimosoideae) is a tree native to Sudan. Habitat: It is distributed along the Nile banks and its tributaries. Constituents: Polyphenolic compounds (ethyle gallate). Traditional use: Gum exudates are used as antidiarrhoetic.

**Talih**: (*Acacia seyal* Del., Fabaceae, subfamily Mimosoideae) is a thorny small tree growing in Sudan and in west and central Africa. Habitat: It is widespread in regions of grass and woodland savanna and grows well on dry cracking clay. The variety *fistula*commonly grows in clay of seasonally wet depressions in central and eastern Sudan (East of the Nile) in deciduous forests. Constituents: The pods contain more than 20% proteins and are very nourishing for livestock. Traditional use: The wood is a fumigant for rheumatic pain. It is also used to protect women from fever after childbirth.

**Tamrind:** (*Tamrindus indica* L., Fabaceae, subfamily Caesalpinioideae) is indigenous to tropical Africa, but is cultivated in India, the East Indies and the West Indies. Habitat: *Tamrindus* grows near valleys and near termite mounds in the savanna in the central and southern parts of Sudan. Constituents: Tamrind contains free and combined organic acids (tartaric, malic, citric) acid potassium tartarate and 25-40% invert sugars. Traditional use: It is used as a gentle laxative and an acid refrigerant. Tamrind also has anti-malaria activity.

## 6 Distribution of Cultivated Medicinal and Aromatic Plants

**Anise, Aniseed:** Aniseed is the fruit of *Pimpinella anisum* L. (Apiaceae). Anise is native to eastern Mediterranean region and was first cultivated in Egypt. It is now widely cultivated in Sudan. Habitat: It grows in light soil. The sowing is done in November and the harvest in April. Constituents: Aniseed contains 3% essential oil (Aniseed oil) with anethole as main component (90%), plus fatty oil, proteins, sugars and organic acids. Traditional use: The steam-distilled oil is an ingredient in carminative and expectorant medicines for children. The greatest quantities of anise however, are used in flavour liqueurs and baking and in perfumery.

**Arghel:** *Solenostemma arghel* (Del.) Hayne (Asclepidaceae) is a herb grows wild in the Egyptian-Sudanese desert. It is also cultivated in Sudan. Habitat: Annual, perennial herb grows in light soils. The best sowing date is between June and July. The harvest occurs in October. It can be cultivated by seeds or cuttings. Constituents: It contains an acidic resin, a glycoside, a bitter principle, choline, phytosterols and amyrine in the lipid fraction. Traditional use: It is used in folk medicine as effective remedy against cough. Infusions of leaves are taken against cramps in gastrointestinal and urinary tracts.

**Black cumin:** (*Nigella sativa* L., Ranunculaceae), is an herbaceous plant growing in countries bordering the Mediterranean Sea. It is extensively cultivated in Sudan. Habitat: An annual herbaceous plant, grown on moderately light soil. The best sowing date is November, the harvest is in April. Constituents: The ripe seeds have a camphor-like scent and bitterness, and also an aromatic taste. Their constituents include saponin, an essential oil, a bitter compound (nigelline) and tannins. Traditional use: It is used in cooking as substituent for pepper. It can be sprinkled on bread and cakes.

**Bishop's weed** is the dried fruit of *Ammi majus* L. (Apiaceae). It is an annual herbaceous plant commonly attaining one meter in height, and grows wild in Egypt and the Mediterranean. It is acclimatized and cultivated both in northern and central Sudan. Habitat: It grows on the Nile bank on different types of soils. The best sowing date is November and the harvest is in May. Constituents: Bishop's weed contains ammoidin, ammidin (imperatorin) and magudin (bergaptene) besides oils and protein. Traditional use: It is used for treatment of leucoderma and skin diseases.

**Capsicum:** (*Capsicum minimum* Roxb., Solanaceae) is an annual or biennial herb indigenous to tropical America, but cultivated in tropical Africa. Habitat: Different species and varieties grow and are being cultivated in the Darfur Province and Kasala Province in Sudan. Constituents: Capsicum contains up to 0.14% pungent principle capsicin, non-pungent alkaloids, starch, oils and vitamin C, especially in the fresh fruit. Traditional use: Capsicum is a stimulant and stomachic substance. It is mainly applied in the form of extracts, tinctures, ointments and plasters to treat rheumatism and sciatica.

**Castor Oil** (*Ricinus communis* L., Euphorbiaceae) is an annual herb in northern and central Europe and in the Mediterranean and a perennial tree in tropical countries. It is native to India and tropical Africa. Habitat: It grows on moderately heavy soils in a wide range of geographical regions in Sudan. It can be cultivated under a variety of climatic conditions. Constituents: Castor beans contain about 50 % oil, which can be extracted by a variety of processes, an extremely toxic albumin (ricin) and the alkaloid ricinine. Traditional use: Medicinally used as a drastic purgative. Also used in cosmetic preparations, lubricant and disinfectant.

**Caraway** (*Carum carvi* L., Apiaceae) is a biennial or perennial herb. It grows throughout Europe and is introduced and cultivated in Sudan. Habitat: It grows on wide range of soil in north and central Sudan. The best sowing date is November; harvest time is in April. Constituents: Its main constituents are essential oils (3-5 %) including carvone and limonene. Further constituents are proteins, starch, sugars and tannins. Traditional use: Caraway has stomachic, antispasmodic, carminative galactogogic, and anthelmintic properties. The fruit is widely used for flavouring foods such as bread, cheese, pickles and sauces.

**Coriander seeds:** Coriander (*Coriandrum sativum* L., Apiaceae) is an annual herb native to southern and eastern Europe. It tolerates a wide range of different climatic conditions and is extensively cultivated in northern Sudan. Habitat: It grows on light soils. The best sowing date is November, and the harvest time is April. Constituents: Good quality seeds contain up to 0.07 % of essential oil. The principle constituent is dextrolinalol. Traditional use: Dried coriander is extensively used for flavouring of sausages, corned beef and similar meat products. Coriander seed can also be distilled to obtain essential oils and oleo resin.

**Cotton:** Oil is obtained from the seeds of several cultivated varieties of *Gossypiumbarbadense* (Malvaceae). It is an annual or biennial shrub. It is indigenous to the West Indies and is now cultivated in Sudan and other tropical and sub-tropical countries. Habitat: It is cultivated on light and heavy soils. The best sowing date is between June and July and the harvest time is in April and May. Constituents: Raw cotton contains 91% cellulose wax, oil, and fat. The oil contains 45% linoleic acid, 30% oleic acid, 20% palmitic acid, 3% stearic acid and 1% stearic and archidic acid. Traditional use: Absorbentcotton is used as protective material in surgery and dressings, filtering medium in pharmacy and as insulating material. The oil is used as solvent for injections and manufacturing of soaps.

**Cumin Seed:** Cumin (*Cuminum cyminum* L., Apiaceae) is an annual herb, which is indigenous to upper Nile regions and is cultivated in countries bordering the Mediterranean Sea. It is extensively cultivated in Sudan. Habitat: It grows on moderate soils in central and northern Sudan. The best sowing date is in November and the harvest time is in April. Constituents: Volatile oils containing cuminic aldehyde. Traditional use: Cumin is a condiment, an agreeable aromatic and is also used in veterinary medicine.

**Fennel:** (*Foeniculm vulgare* Mill., Apiaceae) is native to Sudan. It grows in gardens and as a field crop, but it is commercially cultivated only on a small scale in Sudan. Habitat: It is cultivated as biennial or short-lived perennial herb in small areas in northern and central Sudan. The best sowing date is in November and it is harvested in April. Constituents: The main constituents are essential oils (up to 6%) with anethole and fenchone. Further constituents include fatty oils, proteins, sugars and mucilage. Traditional use: The oil is used in Germany and other European countries for the flavouring of foods and liqueurs. It is also used in the perfumery industry. Pharmaceutically, it is used as aromatic and carminative.

**Fenugreek Seeds:** Fenugreek (*Trigonella foenum-graceum,* Leguminosae/Fabaceae) is an annual herb probably indigenous to an area extending from Iran to northern India, but now it is cultivated in China, as well as northern and eastern Africa. It also grows well in Sudan. Habitat: The plant grows in northern and central Sudan on a wide range of soils. The best sowing date is in November and the harvest is in April. Constituents: The constituents of the seed include oil (about 70%), a mucilage and saponins (1–2%). It also contains proteins and vitamins. Traditional use: Fenugreek seed is generally found in most blends of curry powder, and can also be found in other foods. It also serves as source for sapogenin disogenin.

**Garlic** (*Allium sativum* L., Liliaceae/ Amaryllidaceae) is a perennial herb indigenous to Mediterranean regions with a bulb divided into segments. It is cultivated in northern and central Sudan. Habitat: It grows on relatively light soil. The best sowing date is November and the harvest time is April. Constituents: Garlic contains volatile oil-containing diallyl sulphide and allylpropyl sulphide, the glycoside allin and the enzyme allisin. Traditional use: Garlic is an important antiseptic. It also has hypotensive, anthelmintic, chloretic and expectorant properties. It is used to treat intestinal infection, hypertention, and arteriosclerosis.

**Karkadeh:** Roselle (*Hibiscus sabdariffa,* Malvaceae) grows naturally in southern Sudan and is cultivated in many parts of Sudan. Habitat: It grows on light soil in rain-fed areas. The best sowing datesare in June andJuly, and it is harvested in the rainy season in October and November. In irrigated areas, it can be harvested three times per year from December to January. Constituents: Sepals contain flavonoids, one of which is delphinidin. The plant also contains glycoside hivicin chloride, red pigments (gossipten and hibiscin) and phytosterolin and organic acids (malic, citric, tartaric, ascorbic and hibiscic acids). Seeds also contain high percentages of mucilage (> 60%) and oil. Traditional use: The ripe calyces are used for hot and cold beverages. Medicinally it is used as antispasmodic, hypotensive and antimicrobial agent and for relaxation of the uterine muscle.

**Khella** is the fruit of *Ammi visnaga* L. (Apiaceae), an annual herbaceous plant widely grown in the Mediterranean region. It is cultivated in northern and central Sudan. Habitat: It grows on different types of soils. The best month for sowing is November and the harvest is done in April. Constituents: It contains a bitter principle khellin, visnagin and khellol glycoside, oil and protein. Traditional use: It relaxes smooth muscles and lowers the urethral tonicity. A decoction is used to ease the passage of kidney calculus. It is the source of khellin.

**Henna:** The dried leaves of *Lawsonia inermis* L. (Lytheraceae), a shrub growing in northern Africa and southern Asia. Habitat: It is a perennial shrub cultivated in many parts of Sudan, especially in northern and central Sudan on moderately light soil. It can be cultivated several times a year. Constituents: Henna contains lawasone, tannins, mucilage and fat. Traditional use: It is a dye for the hair, skin and nails. Medicinally it is used as a fungicide. It is also used for perfume and cosmetics as well as soap.

**Sesame oil** is obtained from the seeds of one or more of the cultivated varieties of *Sesamum indicum* L. (Pedaliaceae). It is an annual herb, cultivated as a cash crop in eastern and central Sudan. Habitat: It is grown in eastern and central parts of Sudan. The best sowing datesare in June and July and the oil is harvested in October and November. Constituents: *Sesamum* seed oil is composed of equal amounts of linoleic acid and oleic acid (40%), 9 % palmitic acid and 4 % stearic acid. It contains a phenolic compound known as sesamol which is present in the unsaponifiable fraction of the oil. Traditional use: It is used as a solvent for intramuscular injections. It has nutritive, laxative, demulcent and emollient properties.

**Senna** (*Senna alexandrina* Mill.., Fabaceae, subfamily Caesalpiniaceae) is a perennial herb, known in commerce as Alexandrian senna. It is indigenous to tropical Africa and it grows wild as well as being cultivated in Sudan. Habitat: It grows on wide range of soils all over Sudan. The best sowing dates fall in June and July. It can be harvested two times a year, after autumn in moist areas and after rainfall in irrigated areas. Constituents: The leaves contain sennosides, aloe-emodin and rhein. Traditional use: The water extract of leaves and fruit is taken as a laxative.

## 7 Domestic Trade of Medicinal and Aromatic Plants and Future Prospects

Plants are usually collected from the wild by villagers and brought to local collectors in provincial regions. The collectors establish small compounds in sheds with weighing balances. Their business assets usually consist of small trucks for transport and ready cash to pay for purchases. The villagers are paid after their goods are weighed and examined. The collectors aggregate the products and bring them to wholesale traders in the city. There are two different systems for herbal products: one system for (i) those purchased by exporters and another for (ii) those purchased by traders for consumption by local populations. Most plants consumed locally are directly obtained by rural people from the wild. Some plant products come from different areas of the country, while others have to be imported from another country. This needs to be arranged by the local trading sectors found in *Attareen* Shops. The main wholesale trading centers for these products are located in large cities such as Omdurman, Ummrowaba, and El Obeid.

The main export items in order of importance are: Gum Arabic, Karkadeh, Senna and Henna. There are collectors in the provincial areas who specialize in each of these products and bring them to specialized wholesalers in Omdurman or Port Sudan. The export of gum is a governmental monopoly and collection for this product is more organized. For the other products, exporters rely on large wholesalers in Omdurman or else have their own collection points, which resemble those of the wholesalers. Wholesalers and exporters are increasingly going directly to provisional collection centers and auctions in order to by-pass middlemen and obtain better prices and larger volumes of their products.

The purchased products are usually packed in jute sacks and weighed. The only added value of the product beyond its natural state is cleaning at this stage. The large exporters are always looking for new products. Sudan, as has been stated before, is rich in a wide variety of medicinal and aromatic plants that can be developed for export, offering prospects of great profits.

There is also the potential for an essential oil industry based on the substantial agricultural resources that are presently under-utilized. This would bring higher profits than current exports do, and also serve the local soaps and cosmetics industries well by replacing cheap plant-based products imported from abroad. Additionally, the qualities of the present export products can be improved by standardization and grading. As in the case of most informal traders, there is no record-keeping of stocks or accounts. Hence, the demands from exporters or wholesale traders are difficult to estimate.

## 8 Medicinal and Aromatic Plants for International Trade

About 80% of the world population still depends on medicinal plants for health care. Around 20% of the drugs in modern pharmacopoeias are plant-driven either as pure phytopharmaceuticals extracted from plants or as semi-synthetic derivatives. Annually, people in industrialized countries spend an estimated European Currency Unit of 9.9 billion for phytomedicines. The top selling products are sleeping drugs, sedatives, tonics and products for the treatment of cardiovascular, respiratory and digestive disorders. The phytotherapeutic market in Europe is growing faster than the market for synthetic drugs.

In the Netherlands and United Kingdom, most phytomedicals are sold as food or food supplements without any medical claims in advertisements or packaging. Several other European countries, notably France, Italy and Germany have accepted herbal products for their markets. Worldwide, at least 122 prescription drugs are based on substance found in plants.[41,42] There is much interest in finding new medicinal plant applications before endangered species become extinct.

The interest in health foods and herbal formulations in the USA has increased during the past decade. Americans spend more than 6 billion US dollars annually on nutritional supplements, with 1 billion US dollars on herbs and teas alone. The market for nutritional supplements is growing by 20% each year.

Such estimates for Europe and USA markets constitute bright prospects for medicinal and aromatic plants-producing countries, which supply most of these herbal materials to industrial countries.

Traditionally, Sudan has been a major exporter to different

African, Asian, and European countries since 1952. The plants exported include Senameka (*Senna alexandria)*, Karkadeh (*Hibiscussabdriffa)*, Henna (*Lawsonia inermis)*, Luban (*Boswellia papyrifera)*, Gum talih (*Acaciaseyal),* Camoon (*Cuminmum cyminum*), Shatta (*Capsicum fruitscence*), Kammon Aswad (*Nigella sativa*), Thoom (*Allium sativum*), Kasbra (*Coriandrum sativum*) and Arabic Gum (*Acacia senegal*). Recently, the demand for *Hibiscus sabdariffa*, *Senna alexandria*, *Senna alexandrina* and *Boswellia papyrifera* has increased. Gum Arabic, Karkadeh, Senna, and Gum olibanum are the most important medicinal and aromatic plants for export. Twelve major companies are involved in these export activities. Consequently, new companies and individual exporters have joined the international market. The variety of types and quantities of plant materials exported are expected to increase in the coming years. In addition, standardization and quality control of medicinal plant products will be critical in order to meet international demands.

### Electronic Supplementary Material

Supplementary material is available in the online version of this article at http://dx.doi.org/ 10.1007/s13659-012-0015-2 and is accessible for authorized users.

**Open Access** This article is distributed under the terms of the Creative Commons Attribution License which permits any use, distribution, and reproduction in any medium, provided the original author(s) and source are credited.

## Electronic supplementary material

Supplementary material, approximately 2.03 MB.

## References

[1] Akerele O. (1988). Fitoterapia.

[2] Khalid H. S. (1991). Biological & Phytochemical Studies on *Albizzia anthelmintica*.

[3] Alkhalifa Al Mugtadir (**908–938**), Cited by Hamid MR. “*Gisat Al Dawa Ailm wa Tahdyat*” (Arabic), MR Hamid, Maktaba Al Academia 121 Shari Al Tahrir Dougi Algeeza, Cairo, Egypt. www.newadvent.org/cathen/10122a.htm.

[4] Abu Bakr Al Razi (**854–932**) *Kitab Alhawi* (Arabic). Ministry of Education Printing Press, Haiderabad, India.

[5] Ibn El Bitar (**1248** A.D.). *Al Jami Li Mfradat Al Dawiyya wa Al Aghzhiyya (Mufradat Ibn EL Bitar) (Arabic)*. Four Volumes, Cairo Matba’at Bulaq, **1874**.

[6] Al Dainori, Ahmed Ibn Daoud. *Kitab Alnabat* (Arabic). Lieden: B. Loin, **1953**.

[7] Daoud Al-Antaky (**1600** A.D.). *Tazkirt Ulil Al Albab wa Aljami’a Lil Ajab Alujab* (Popularly known as Tazkirt Daoud Alantaki) (Arabic). Cairo; Matha’at Abd Alraziq publishers.

[8] Khalid S. A. (2009). Nat. Prod. Comm..

[9] El-Hamidi A. (1970). Planta Med..

[10] Banthorpe D. V., Duprey R. J., Hassan M., Janes J. F., Modawi B. M. (1976). Planta Med..

[11] Antoun M. D., El Khawad A. O., Taha O. M. (1977). Lloydia.

[12] Antoun M. D., Taha O. M. (1981). J. Nat. Prod..

[13] El Sheikh M. O., El Hassan G. M., El Tayeb Abdel Hafeez A. R., Abdalla A. A., Antoun M. D. (1982). Planta Med..

[14] El Ghazali G. E. B. (1986). Medicinal Plants of Sudan Part I. Medicinal Plants of Erkawit.

[15] El Ghazali G. E. B., Bari E. A., Bashir A. K., Salih A. M. (1987). Medicinal Plants of Sudan Part II. Medicinal Plants of Eastern Nuba Mountains.

[16] El Ghazali G. E. B., El Tohami M. S., El Egami A. A. B. (1994). Medicinal Plants of the Sudan Part III, Medicinal Plants of White Nile Province.

[17] El Ghazali G. E. B., El Tohami M. S., El Egami A. A., Abdalla W. E., Galal M. (1997). Medicinal Plants of the Sudan Part IV. Medicinal Plants of North Kordofan.

[18] El Ghazali G. E. B., Abdalla W. E., Khalid H. E., Khalafalla M. M., Hamad A. A. (2003). Medicinal Plants of the Sudan Part V. Medicinal Plants of Ingassana Area.

[19] El-Tahir A., Satti G. M., Khalid S. A. (1999). Phytother. Res..

[20] Hussein G., Miyashiro H., Nakamura N., Hattori M., Kakiuchi N., Shimotohno K. (2000). Phytother. Res..

[21] Elegami A. A., Almagboul A. Z., Omer M. E., El Tohami M. S. (2001). Fitoterapia.

[22] Ali H., König G. M., Khalid S. A., Wright A. D., Kaminsky R. (2002). J. Ethnopharmacol..

[23] Ahmed E.-H. M., Nour B. Y., Mohammed Y. G., Khalid H. S. (2010). Environmental Health Insights.

[24] El Kheir Y. M., El Tohami M. S. (1979). J. Trop. Med. Hygiene.

[25] Ahmed E. H., Bashir A. K., El Kheir Y. M. (1984). Planta Med..

[26] Koko W. S., Galal M., Khalid H. S. (2000). J. Ethnopharmacol..

[27] Koko W. S., Abdalla H. S., Galal M., Khalid H. S. (2005). Fitoterapia.

[28] Koko W. S., Mesaik M. A., Yousaf S., Galal M., Choudhary M. I. (2008). J. Ethnopharmacol..

[29] White F. (1983). Nat. Resources Res..

[30] De Silva T. (1996). Manual on The Essential Oil Industry, Presentations made by the resource persons at third UNIDO workshop on essential oil and aroma chemical industries held at the Andoulu University, Medicinal and Aromatic Plant and Drug development.

[31] Arcury, T. A.; Grzywacz, J. G.; Stoller, E. P.; Bell, R. A.; Altizer, K. P.; Chapman, C.; Quandt, S. A. *J. Gerontology* **2009**, Series B, *Psychol. Sci. and Social Sci. 64*, 635–643. 10.1093/geronb/gbp011 PMC2728088 19289376

[32] Bakhiet A. O., Adam S. E. (1995). Veterinary Hum. Toxicol..

[33] Omer S. A., Adam S. E. (1999). Veterinary Hum. Toxicol..

[34] Barri M. E., Onsa T. O., Elawad A. A., Elsayed N. Y., Wasfi I. A., Abdul-Bari E. M., Adam S. E. (1983). J. Comparative Pathol..

[35] Barakat S. E., Wasfi I. A., Adam S. E. (1983). Veterinary Pathol..

[36] Galal M., Adam S. E. (1988). Veterinary Hum. Toxicol..

[37] Galal M., Bashir A. K., Salih A. M., Adam S. E. (1991). J. Ethnopharmacol..

[38] Galal M., Bashir A. K., Salih A. M., Adam S. E. (1991). Veterinary Hum. Toxicol..

[39] Baser K. H. C., Abeywickrama K. (1995). Fact-Finding and Preparatory Assistance to Assess the Potential for the Industrial Utilization of Medicinal and Aromatic Plants in Sudan.

[40] Vasisht, K.; Kumar, V. *Compendium of Medicinal and Aromatic Plants* — Volume I — Africa. UNIDO (United Nations Industrial Development Organization), **2006**.

[41] Farnsworth N. R., Akerele O., Bingel A. S., Soejarta D. D., Eno Z. (1985). Bull. World Health Org..

[42] Blumenthal M. (2003). The ABC Clinical Guide to Herbs.

[43] Singh R., Singh B., Singh S., Kumar N., Kumar S., Arora S. (2008). Toxicol. In Vitro.

[44] Singh R., Singh B., Singh S., Kumar N., Kumar S., Arora S. (2010). J. Food Chem..

[45] Eldeen I. M., Van Heerden F. R., Van Staden J. (2010). J. Ethnopharmacol..

[46] Aspinall G. O., Hunt K., Morrison I. M. (1966). J. Chem. Soc. Perkin 1.

[47] Mueller-Harvey I., Hartley R. D., Reed J. D. (1987). J. Sci. Food Agricult..

[48] Mangan J. L. (1988). Nutr. Res. Rev..

[49] Kinghorn A. D., Chai H. B., Sung C. K., Keller W. J. (2011). Fitoterapia.

[50] Viljoen A. M., Wyk B. V., Newton L. E. (2001). Biochem. Systematics Ecol..

[51] Mahmoud A. A., Ahmed A. A., El Bassuony A. A. (1999). Fitoterapia.

[52] Siddiqui B. S., Afshan F., Gulzar T., Hanif M. (2004). Phytochem..

[53] Ragasa C. Y., Nacpil Z. D., Natividad G. M., Tada M., Coll J. C., Rideout J. A. (1997). Phytochem..

[54] Beit-Yannai E., Ben-Shabat S., Goldschmidt N., Chapagain B. P., Liu R. H., Wiesman Z. (2011). Phytochem. Lett..

[55] Kim T. R., Pastuszyn A., Vanderjagt D. J., Glew R. S., Millson M., Glew R. H. (1997). J. Food Comp. Analysis.

[56] Hamm S., Bleton J., Connan J., Tchapla A. (2005). Phytochemistry.

[57] Schweiggert U., Carle R., Schieber A. (2006). Analyt. Chim. Acta..

[58] Tannin-Spitz T., Grossman S., Dovrat S., Gottlieb H. E., Bergman M. (2007). Biochem. Pharmacol..

[59] Ngadjui B. T., Abegaz B. M., Keumedjio F., Folefoc G. N., Kapche G. W. F. (2002). Phytochemistry.

[60] Ngadjui B. T., Folefoc G. G., Keumedjio F., Dongo E., Sondengam B. L., Connolly J. D. (1999). Phytochemistry.

[61] Block S., Baccelli C., Tinant B., Meervelt L. V., Rozenberg R., Jiwan J. L. H., Llabrès G., De Pauw-Gillet M. C., Quetin-Leclercq J. (2004). Phytochemistry.

[62] Stange R. R., Sims J. J., Midland S. L., McDonald R. E. (1999). Phytochemistry.

[63] Shahi A. K., Kaul M. K., Gupta R., Dutt P., Chandra S., Qazi G. N. (2005). Flavour Fragrance J..

[64] El-Askary H. I., Meselhy M. R., Galal A. M. (2003). Molecules.

[65] Ragasa C. Y., Co A. L., Rideout J. A. (2005). Nat. Prod. Res..

[66] *The Ayurvedic Pharmacopoeia of India*. Part I, Vol. II. Government of India, Ministry of Health and Family Welfare.Namo Narayana. First edition, New Delhi, India, **1999**.

[67] Bouchet N., Levesque J., Blond A., Bodo B., Pousset J. L. (1996). Phytochemistry.

[68] Mahmoud E. H. N., Khalid S. A. (1997). Phytochemistry.

[69] Fiot J., Sanon S., Azas N., Mahiou V., Jansen O., Angenot L., Balansard G., Ollivier E. (2006). Ethnopharmacol..

[70] Tseng T. H., Hsu J. D., Lo M. H., Chu C. Y., Chou F. P., Huang C. L., Wang C. J. (1998). Cancer Lett..

[71] Hou D. X., Tong X., Terahara N., Luo D., Fujii M. (2005). Arch. Biochem. Biophys..

[72] Abdelgaleil S. A. M., Okamura H., Iwagawa T., Sato A., Miyahara I., Doe M., Nakatani M. (2001). Tetrahedron.

[73] Govendachari T. R., Krishna Kumari G. N. (1998). Phytochemistry.

[74] Hsouna A. B., Trigui M., Culioli G., Blache Y., Jaoua S. (2011). Food Chem..

[75] Erdelmeier C. A., Regenass U., Rali T., Sticher O. (1992). Planta Med..

[76] Shigemori H., Kagata T., Ishiyama H., Morah F., Ohsaki A., Kobayashi J. (2003). Chem. Pharm. Bull..

[77] Grayer R. J., Kite G. C., Veitch N. C., Eckert M. R., Marin P. D., Senanayake P., Paton A. J. (2002). Biochem. Syst. Ecol..

[78] Baliga M. S., Baliga B. R. V., Shaun Mathew Kandathil S. M., Bhat H. P., Vayalil P. K. (2011). Food Res. Intern..

[79] Ferraz A. C., Angelucci M. E., Da Costa M. L., Batista I. R., De Oliveira B. H., Da Cunha C. (1999). Pharmacol. Biochem. Behav..

[80] Holfelder M. G. A. H., Steck M., Komor E., Seifert K. (1998). Phytochemistry.

[81] Malik S., Ahmad S. S., Haider S. I., Muzaffar A. (1987). Tetrahedron Lett..

[82] Ohtani K., Kasai R., Yamasaki K., Tanaka O., Kamel M. S., Assaf M. H., El-Shanawani M. A., Ali A. A. (1992). Phytochemistry.

[83] Oshio H., Imai S., Fujioka T., Sugawara M., Miyamoto M., Tsukui M. (1974). Chem. Pharm. Bull..

[84] Oshio H., Tsukui M., Matsuoka T. (1978). Chem. Pharm. Bull..

[85] Wu Q. P., Wang Z. J., Fu M. H., Tang L. Y., He Y., Fang J., Gong Q. F. (2007). Zhong Yao Cai.

[86] Hasan A. F., Furumoto T., Begum S., Fukui H. (2001). Phytochemistry.

[87] Furumoto T., Iwata M., Feroj Hasan A. F., Fukui H. (2003). Phytochemistry.

[88] Eltayeb E. A., Al-Ansari A. S., Roddick J. G. (1997). Phytochemistry.

[89] Kamel M. S., Ohtani K., Hasanain H. A., Mohamed M. H., Kasai R., Yamasaki K. (2000). Phytochemistry.

[90] Hassan H. A., Hamed A. I., El-Emary N. A., Springue I. V., Mitome H., Miyaoka H. (2001). Phytochemistry.

[91] Sudjaroen Y., Haubner R., Würtele G., Hull W. E., Erben G., Spiegelhalder B., Changbumrung S., Bartsch H., Owen R. W. (2005). Food Chem. Toxicol..

[92] Han Y., Nishibe S., Noguchi Y., Jin Z. (2001). Phytochemistry.

[93] Evidente A., Fernández-Aparicio M., Andolfi A., Rubiales D., Motta A. (2007). Phytochemistry.

