# Supplementary material for: Gems from traditional north-African medicine: medicinal and aromatic plants from Sudan
Source: Nat Prod Bioprospect. 2012 Apr 17;2(3):92–103. doi: 10.1007/s13659-012-0015-2 (PMC4131591; doi:10.1007/s13659-012-0015-2)
Supplement: Supplementary file 1 — Supplementary material, approximately 2.03 MB. [file 13659_2012_15_MOESM1_ESM.pdf]

Electronic Supplementary Material

Gems from traditional north-African medicine: medicinal and aromatic plants from Sudan

Hassan KHALID,[a] Wail Elsadig ABDALLA,[a] Haider ABDELGADIR,[a] and Thomas EFFERTH[b,]*

[a]The Medicinal and Aromatic Plants Research Institute (MAPRI), National Centre for Research, Mac Nimr Street, Khartoum, Sudan

[b]Department of Pharmaceutical Biology, Institute of Pharmacy and Biochemistry, Johannes Gutenberg University, Staudinger Weg 5, 55128 Mainz, Germany

Received 15 February 2012; Accepted 29 March 2012

Abstract: Sudanese folk medicine represents a unique blend of indigenous cultures with Islamic, Arabic and African traditions. In addition, Sudan encompasses different terrains and climatic zones, ranging from desert and semi-desert in the north to equatorial with a short rainy season (semi-aridand semi-humid) in the centre to equatorial with a long rainy season (arid-humid and equatorial-humid) in the south. This variation contributes to the immense diversity of vegetation in the region. The flora of Sudan consists of 3137 species of flowering plants belonging to 170 families and 1280 genera. It is estimated that 15% of these plants are endemic to Sudan. The intersection of diverse cultures and the unique geography holds great potential for Sudanese herbal medicine. Medicinal and aromatic plants and their derivatives represent an integral part of life in Sudan. Indigenous remedies are the only form of therapy available to the majority of poor people. It has been estimated that only 11% of the population has access to formal health care. Therefore, research on the desired pharmacological effects and possible unwanted side effects or toxicity is required to improve efficacy and safety of Sudanese herbal medicine. In the future, it would be preferable to promote the use of traditional herbal remedies by conversion of raw plant material into more sophisticated products instead of completely replacing the traditional remedies with synthetic products from industrialized countries. The present review gives an overview of traditional Sudanese medicinal and aromatic herbs and their habitats, traditional uses, and phytochemical constituents.

*To whom correspondence should be addressed. E-mail: efferth@uni-mainz.de

Acacia nilotica:

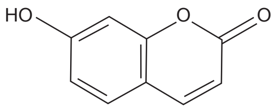

umbelliferone

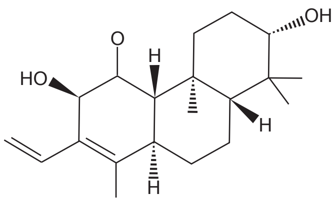

niloticane

Acacia seyal:

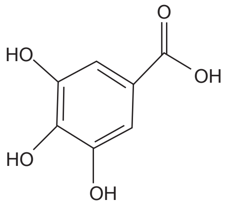

gallic acid

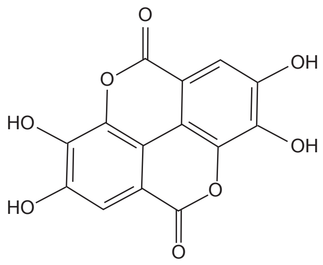

ellagic acid

Acacia senegal:

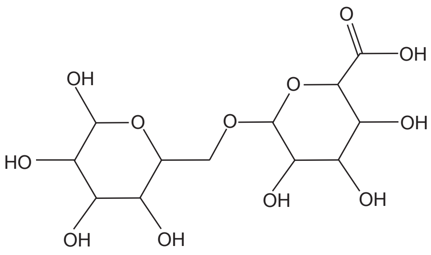

6-O-D-glucopyranuronosyl
-D-galactose

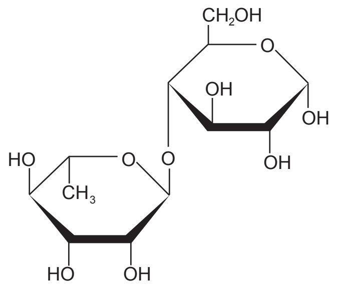

4-O-L-rhamnopyransoyl
α-D-glucose

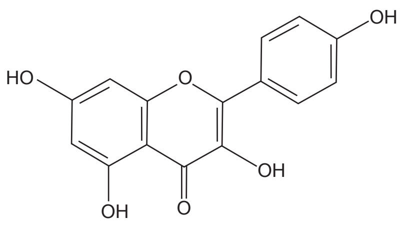

kaempferol

Adansonia digitata:

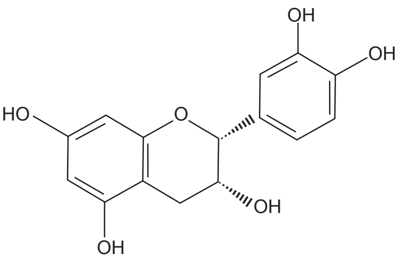

epicatechin

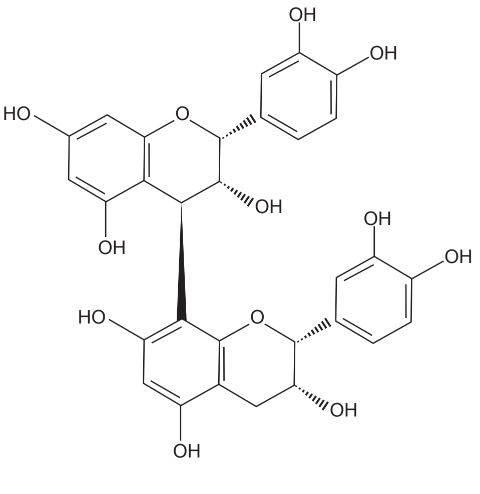

procyanidin B2

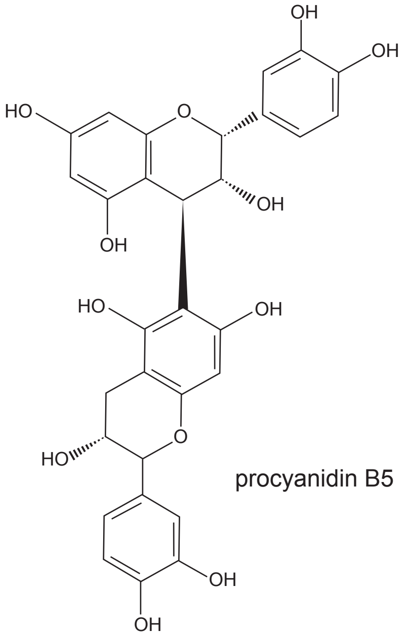

procyanidin B5

Aloe sinkatana:

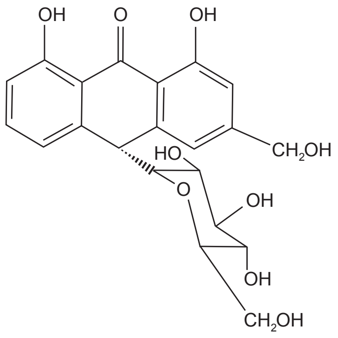

aloin

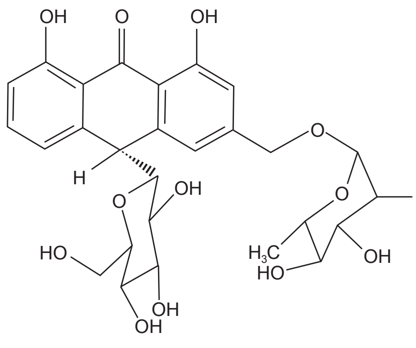

aloinoside

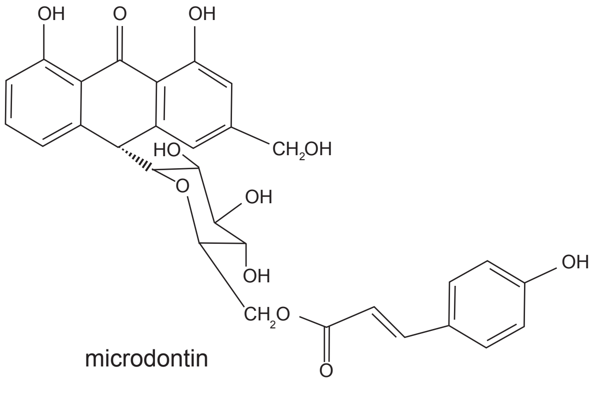

microdontin

Ambrosia maritima:

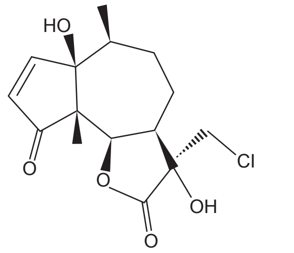

(3R,3aR,6S,6aR,9aS,9bR)-3-(chloromethyl)-3,3a,4,5,6,6a,9a,9b-octahydro-3,6a-dihydroxy-6,9a-dimethylazuleno[4,5-b]furan-2,9,-dione

Azadirachta indica:

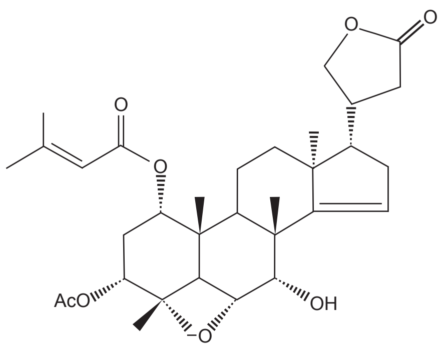

azadirachtolide

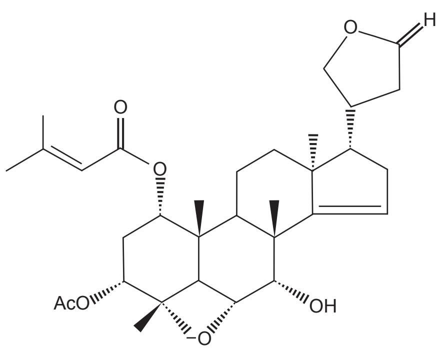

deoxyazadirachtolide

Balanites aegyptiaca:

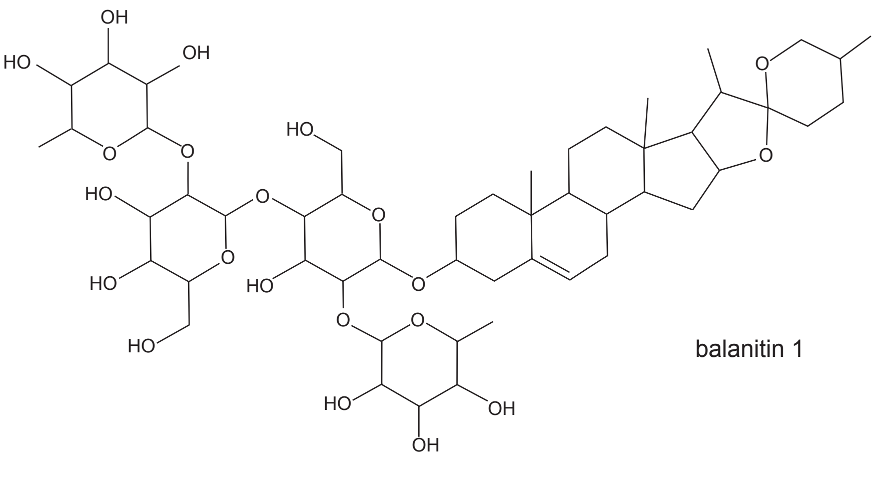

balanitin 1

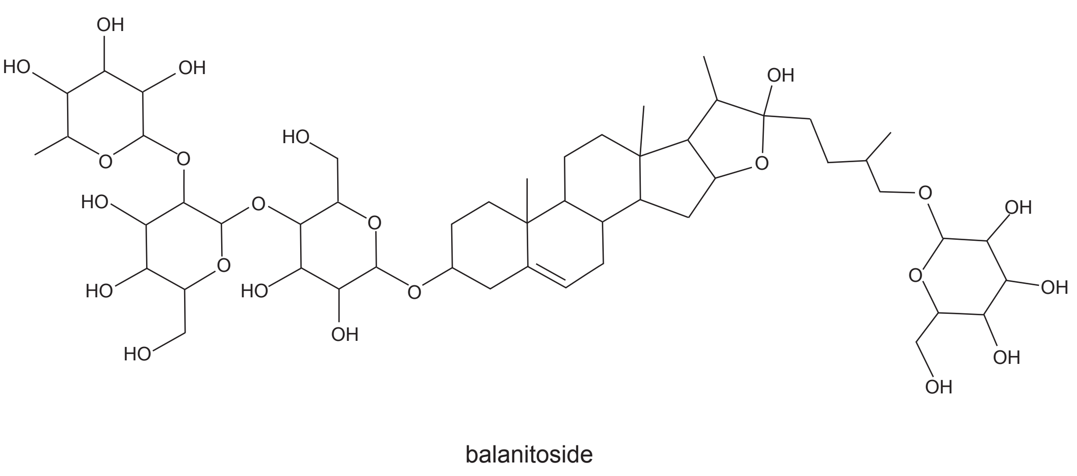

balanitoside

Boscia senegalensis:

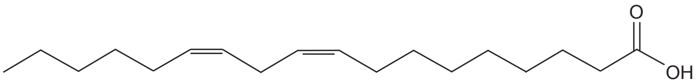

linoleic acid

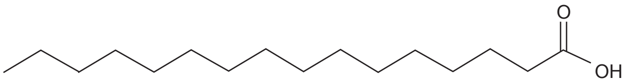

palmitic acid

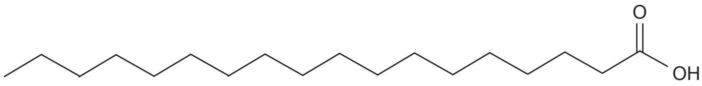

stearic acid

Boswellia payrifera:

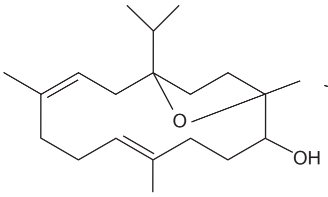

incensole

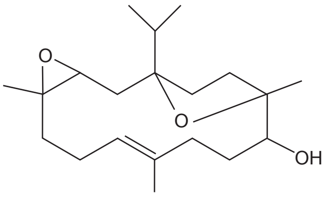

incensole oxide

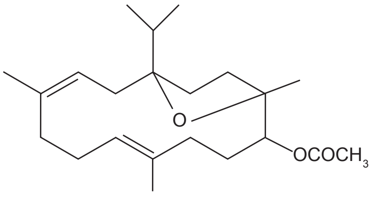

incensole acetate

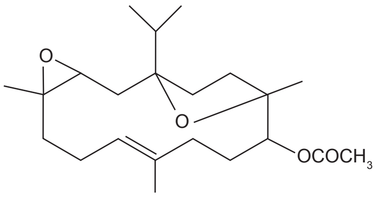

incensole oxide acetate

Capsicum frutescens, Citrullus colocynthis:

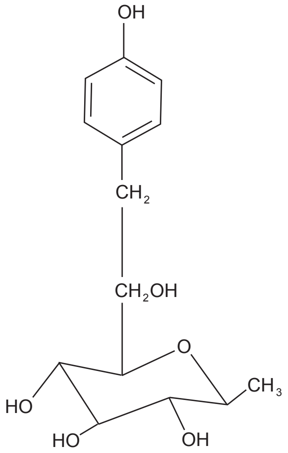

C-p-hydroxybenzoylglycoflavones

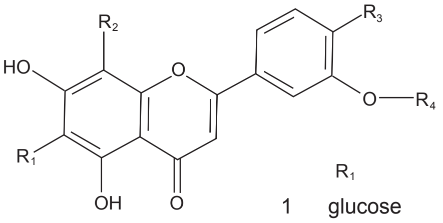

	R_1	R_2	R_3	R_4
1	glucose	H	OMe	H
2	glucose	H	OH	H
3	glucose	H	H	H
4	glucose	p-hydroxybenzyl	H	H
5	p-hydroxybenzyl	glucose	H	H
6	glucose	p-hydroxybenzyl	H	glucose

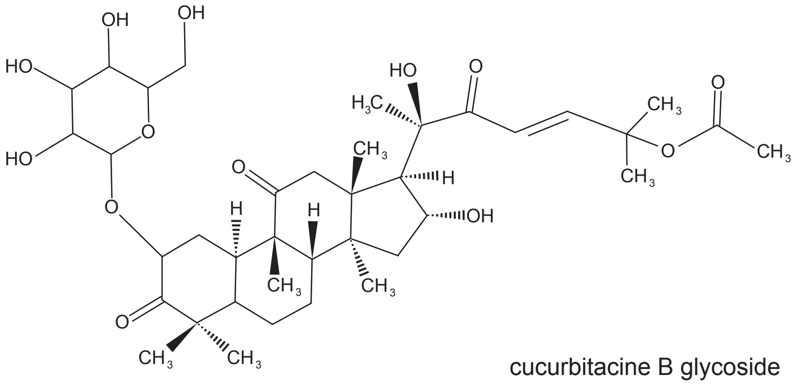

cucurbitacine B glycoside

Croton zambiscus:

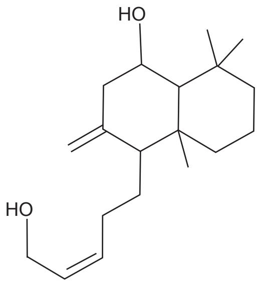

crotonadiol

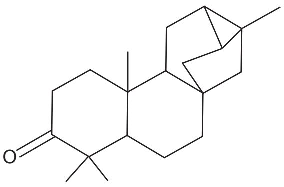

3-trachylobanone

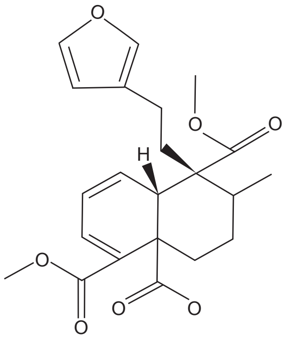

crotozamberfuran B

Cucurbita maxima:

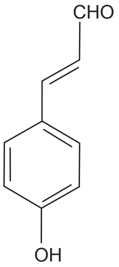

trans-p-coumaryl aldehyde

Cymbopogon communatus:

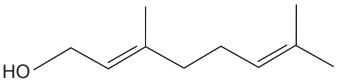

geraniol

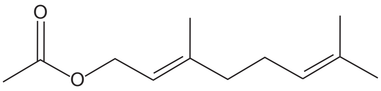

geranyl acetate

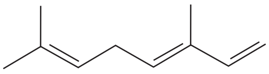

ocimene

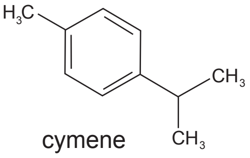

cymene

Cymbopogon nervatus, Cymbopogon proximus:

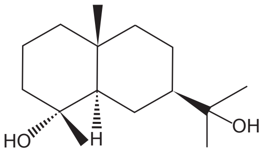

cryptomeridiol, proximadiol

Grewia tenax:

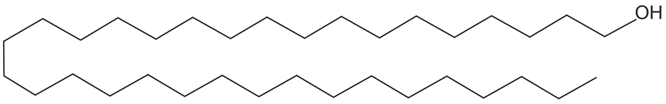

tricontan-1-ol

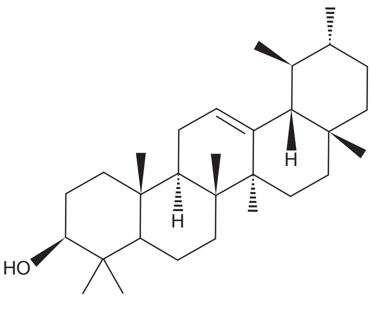

α-amyrine

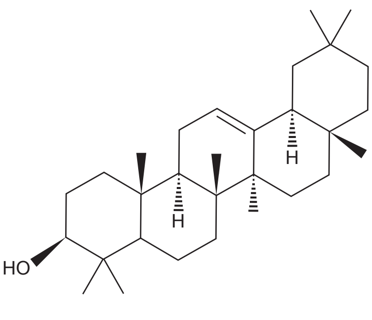

β-amyrine

Guiera senegalensis:

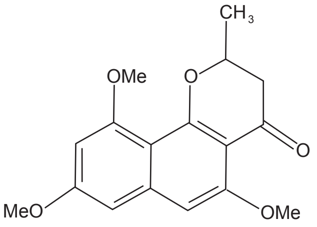

5-methyldihydro-
flavasperone

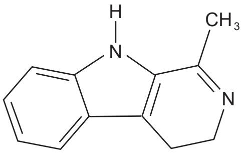

harmalan

Hibiscus sabdariffa:

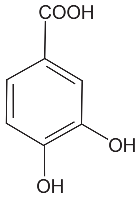

protocatechuic
acid

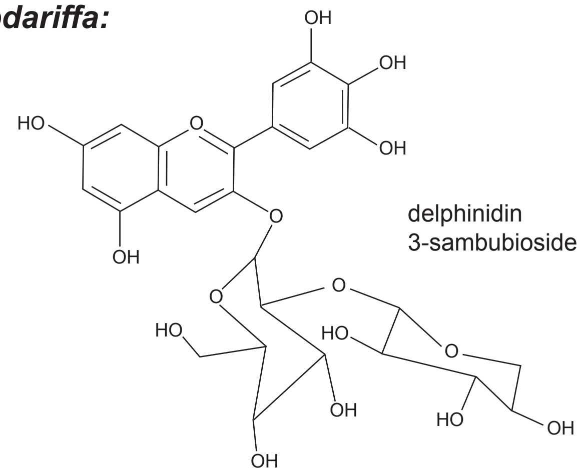

delphinidin
3-sambubioside

Khaya senegalensis:

khayanolide A

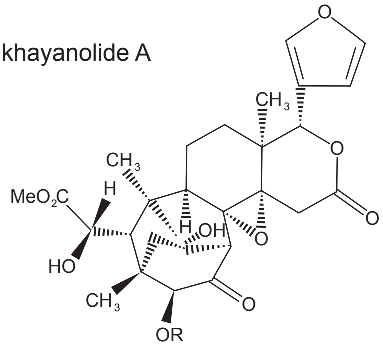

khayanolide B

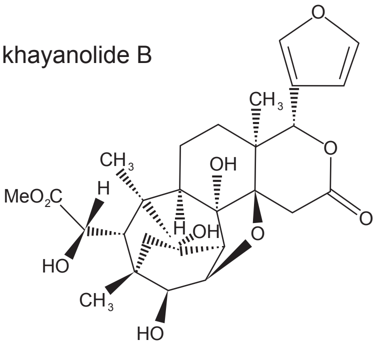

khayanolide C

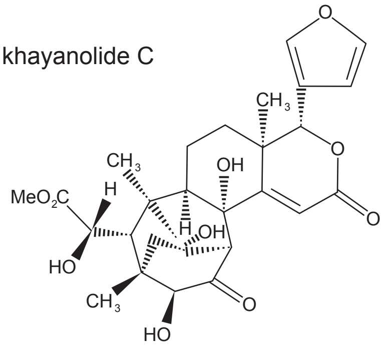

seneganolide

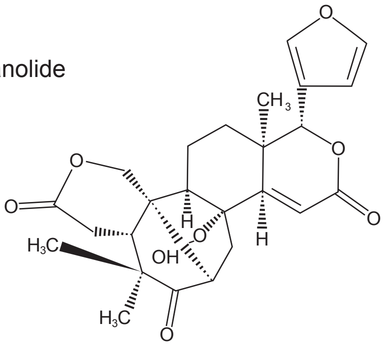

6-deoxydestigloylswietenine

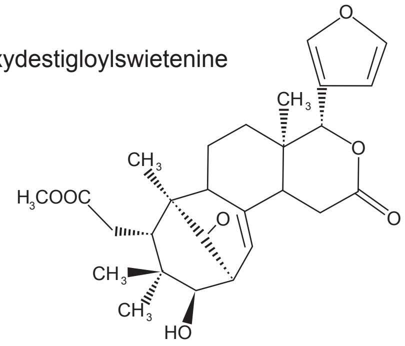

Lawsonia inermis:

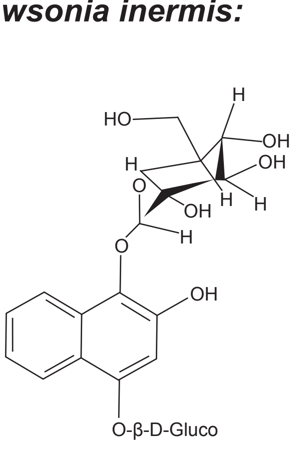

lawsoniaside

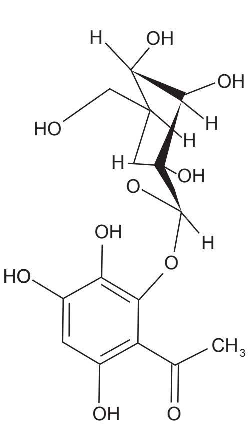

lalioside

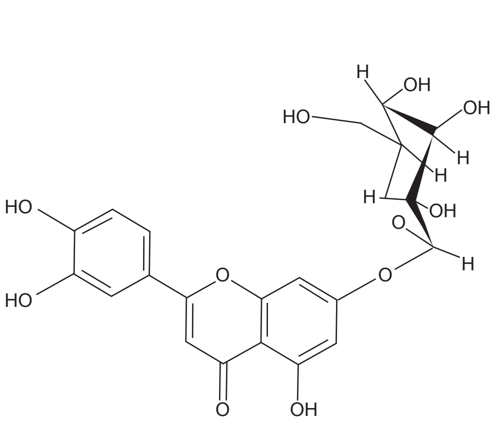

luteolin-7-β-D-glucopyranoside

Nauclea latifolia:

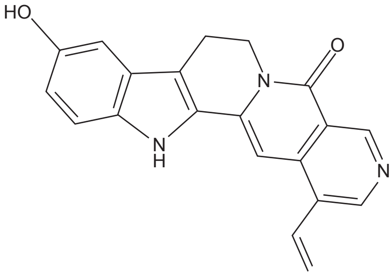

10-hydroxyangustine

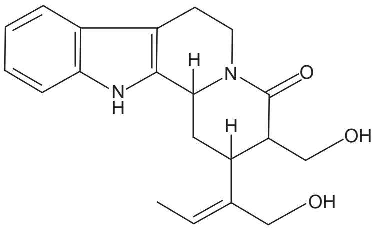

naucleamide A

Ocimum basilicum:

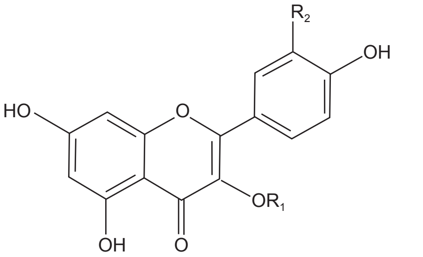

R1	R2	compound
Xyl(1’’’→2’’)Gal	OH	quercetin 3-O-xylosyl(1’’’→2’’)galactoside
Gha(1’’’→6’’)Glc	OH	quercetin 3-O-rutinoside
Glc	OH	quercetin 3-O-glucoside
(6’’-O-malony)Glc	OH	quercetin 3-O-(6’’ -Omalony)glucoside
Rha(1’’’→6’’)Glu	H	kaempferol-3-O-rutinoside
Glc	H	kaempferol-3-O-glucoside
(X’’-O-malony)Glc	H	kaempferol-3-O-malonylglucoside

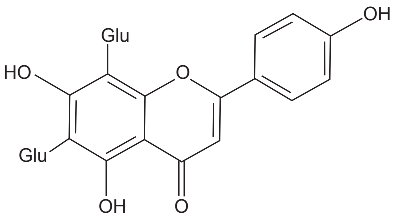

vicenin-2

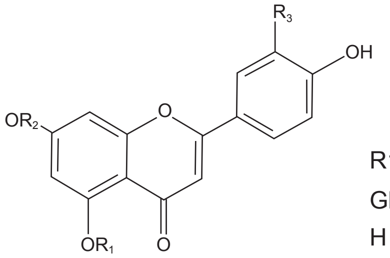

R1	R2	R3	compound
Glu	H	OH	luteolin 5-O-glucoside
H	Gluc	OH	luteolin 7-O-glucoside
H	Gluc	H	apigenin 7-O-glucoside

Phoenix dactylifera:

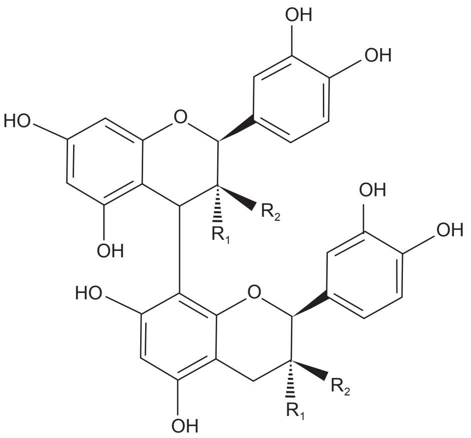

procyanidin (4→8) dimer

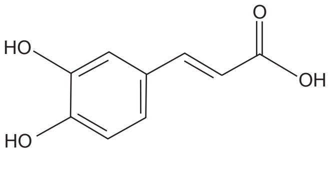

caffeic acid

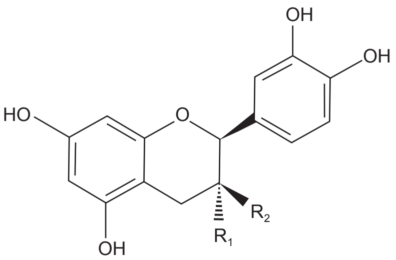

R1	R2	compound
H	OH	catechin
OH	H	epicatechin

Ricinus communis:

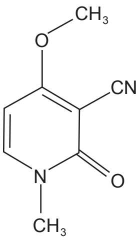
ricinin

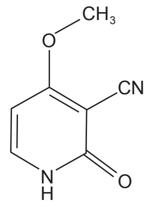
N-demethyl-
ricinin

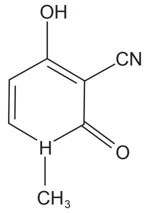
O-demethyl-
ricinin

Salvadora persica:

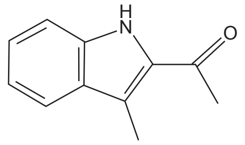
1-(3-methyl-1H
-indol-2-yl)ethanone

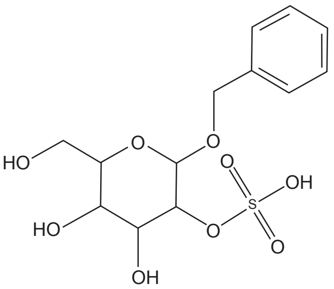
benzylglucoside
-2-O-sulfate

Senna alexandrina:

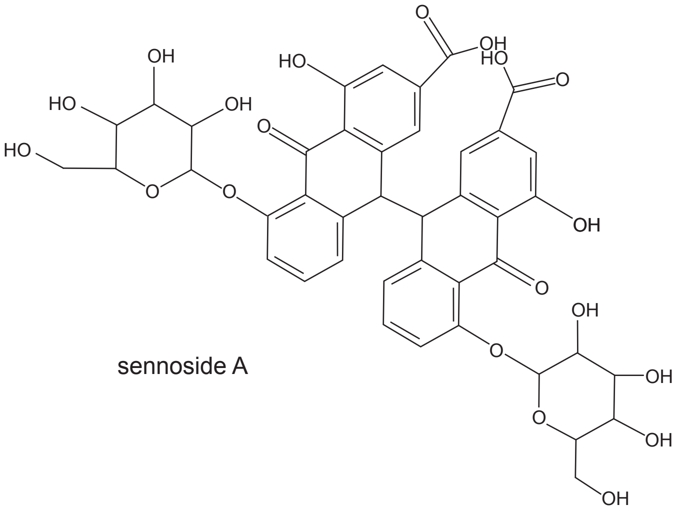
sennoside A

Solanum nigrum:

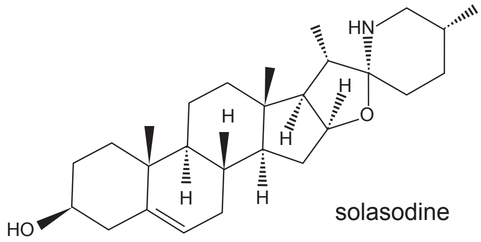
solasodine

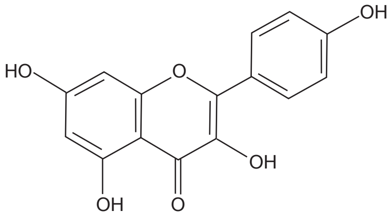
kaempferol

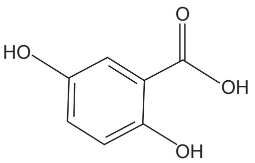
2,5-dihydroxy-
benzoic acid

Sesamum indicum:

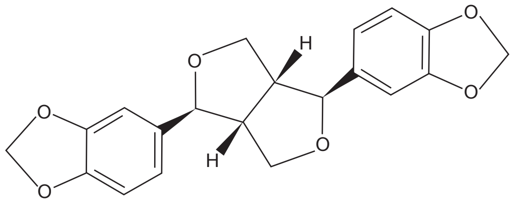
sesamin

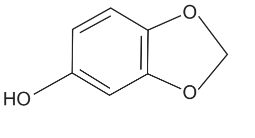
sesamol

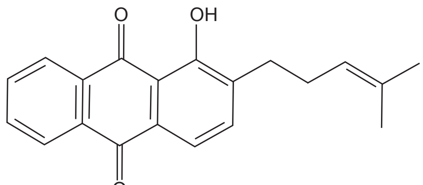
anthrasesamone A

Solenostemma argel:

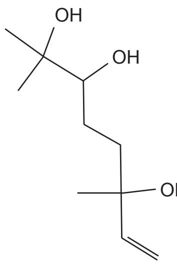

-6,7dihydroxydihydrolinalool

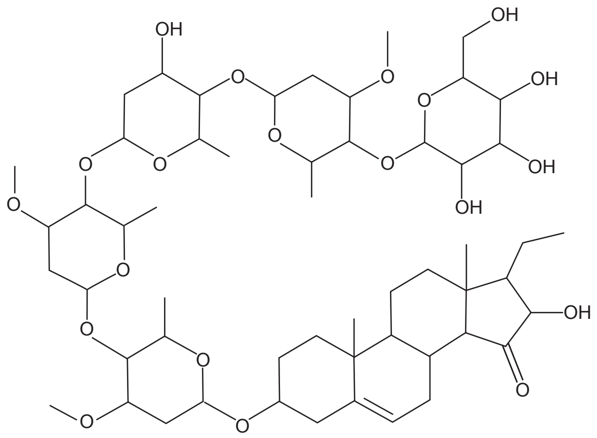

stemmoside G

Tamarindus indica:

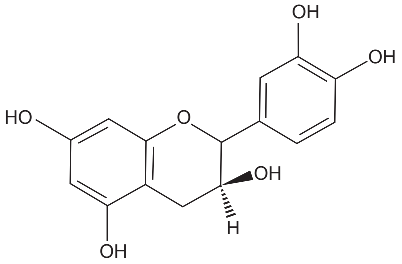

(+)-catechin

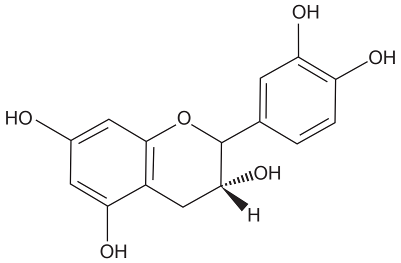

(-)-epicatechin

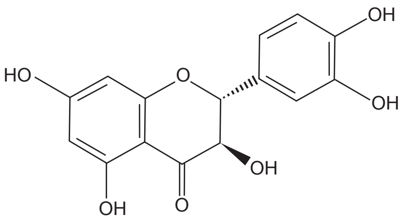

taxifolin

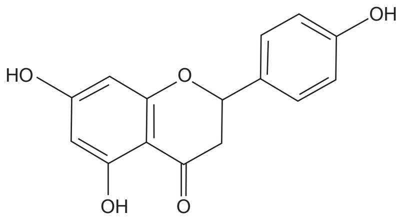

naringenin

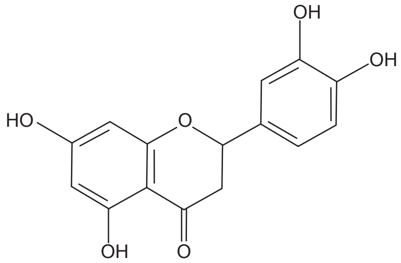

eriodictyol

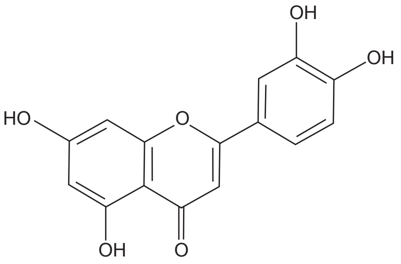

luteolin

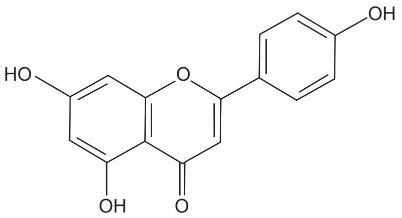

apigenin

Trigonella foenum-graecum:

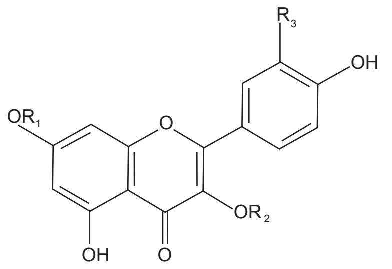

R1	R2	R3	compound
H	Glc(1-2)gal	H	kaempferol 3-O-β-D-glucopyranosyl)-β-D-galactopyranoside
Glc	Glc(1-2)gal	H	kaempferol-3-O-(2-O-β-D-glucopyranosyl)-β-D-galactopyranoside-7-O-β-D-glucopyranoside
Glc(1-2)(6''-Ac)gal		H	kaempferol 3-O-(2-O-β-D-glucopyranosyl)-(6''-O-acetyl)-β-D-galactopyranoside-7-O-β-D-glucopyranoside
Glc	Glc(1-2)gal	OH	quercetin 3-O-(2-β-D-glucopyranosyl)-β-D-galactopyranoside-7-O-β-D-glucopyranoside

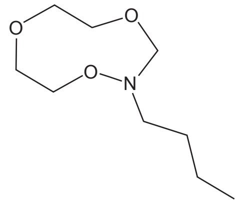

trigoxazonane
